# The Budding Yeast Amphiphysin Complex Is Required for Contractile Actin Ring (CAR) Assembly and Post-Contraction GEF-Independent Accumulation of Rho1-GTP

**DOI:** 10.1371/journal.pone.0097663

**Published:** 2014-05-29

**Authors:** Michael John Cundell, Clive Price

**Affiliations:** School of Health and Medicine, Division of Biomedical and Life Sciences, Lancaster University, Lancaster, United Kingdom; Newcastle University, United Kingdom

## Abstract

The late events of the budding yeast cell division cycle, cytokinesis and cell separation, require the assembly of a contractile actomyosin ring (CAR), primary and secondary septum formation followed by enzymatic degradation of the primary septum. Here we present evidence that demonstrates a role for the budding yeast amphiphysin complex, a heterodimer comprising Rvs167 and Rvs161, in CAR assembly and cell separation. The *iqg1-1* allele is synthetically lethal with both *rvs167* and *rvs161* null mutations. We show that both Iqg1 and the amphiphysin complex are required for CAR assembly in early anaphase but cells are able to complete assembly in late anaphase when these activities are, respectively, either compromised or absent. Amphiphysin dependent CAR assembly is dependent upon the Rvs167 SH3 domain, but this function is insufficient to explain the observed synthetic lethality. Dosage suppression of the *iqg1-1* allele demonstrates that endocytosis is required for the default cell separation pathway in the absence of CAR contraction but is unlikely to be required to maintain viability. The amphiphysin complex is required for normal, post-mitotic, localization of Chs3 and the Rho1 GEF, Rom2, which are responsible for secondary septum deposition and the accumulation of GTP bound Rho1 at the bud neck. It is concluded that a failure of polarity establishment in the absence of CAR contraction and amphiphysin function leads to loss of viability as a result of the consequent cell separation defect.

## Introduction

Cytokinesis is the final stage in the mitotic cell division process whereby cell separation is effected to generate two daughter cells. In yeast and animal cells this requires the assembly of a contractile actin ring (CAR) that initiates membrane ingression followed by membrane scission [1]. These events are necessarily coupled to the completion of mitosis and contraction initiates immediately after mitotic spindle breakdown [2]. In budding yeast, *Saccharomyces cerevisiae*, CAR activity is also coupled to septation and the deposition of new cell wall material [3], [4], [5]. Septation requires the activity of two chitin synthases, Chs2 and Chs3, governing the synthesis of the primary and secondary septa respectively [6]. Secondary septum formation also requires glucan synthase activities [7]. Following successful construction of the primary and the flanking secondary septa the primary septum is removed by the activity of chitinase to allow the completion of cell separation [8].

Many components of the CAR are conserved between yeast and higher eukaryotes, most obviously actin (Act1) and type II myosin (Myo1) and yeast serves as an attractive model in which to study the requirements for CAR assembly and contraction. In *S. cerevisiae* a central regulator of CAR structure and function is Iqg1p, the single IQGAP homologue. *IQG1* is an essential gene and mutants fail to assemble a CAR [9], [10]. A large number of proteins involved in CAR function and cell separation have been described [2] and of particular relevance here are Hof1, Cyk3 and Inn1 [11], [12], [13]. Various interactions between these proteins and dependency relationships determining the order of bud neck recruitment have been described [10], [14], [15], [16], [12], [13], [17], [18], [19], [20], [21], [22]. CAR assembly is also dependent upon the Rho1 GTPase, which is recruited to the division site through interaction with the Tus1/Rom2 guanine nucleotide exchange factors (GEFs) during mitosis. Subsequently Rho1-GTP accumulates at the bud neck via a second mechanism requiring GEF independent interaction between the polybasic C-terminus of Rho1 and the plasma membrane [23], [24], [25]. More recently activation of Rho1 has been shown to be required for secondary septum formation but not for CAR contraction or cleavage furrow ingression [26].

A second major role for actin in *S. cerevisiae* is in endocytosis and the establishment of polarized cell growth [27]. CAR assembly requires a re-organization of the actin cytoskeleton with a switch from a polarized growth and endocytosis associated pattern to actin incorporation into the CAR at the bud neck [28], [5]. To date only one additional component of the endocytic pathway, Bsp1, has been implicated in CAR assembly although its function in either cytokinesis or endocytosis remains to be fully elucidated [29]. The requirement for membrane curvature, assembly of protein complexes at sites of membrane deformation and membrane scission are common to both endocytosis and cytokinesis. In the case of endocytosis this leads to vesicle formation and internalization at the plasma membrane and a vast body of work has led to the proposal of a convincing model for this process in yeast [30], [31]. A central player in this model is the heteromeric yeast amphiphysin complex, containing Rvs167 and Rvs161. These proteins belong to a family of BAR domain proteins, both members of the sub-family of N-BAR containing proteins. Extensive physical and genetic analysis has demonstrated that the BAR domain is able to bind to the plasma membrane structures and act to develop membrane curvature [32], [33]. This property is thought to underpin the role of the yeast amphiphysin complex in endocytosis and by extension to be the key property and functional activity of all amphiphysins [33]. In addition to defects in endocytosis *rvs167* and *rvs161* mutations confer salt sensitivity and actin cytoskeleton polarity perturbation [34], [35], [36], [37].

Several studies have demonstrated that genetic interactions between genes involved in CAR and cell separation functions are manifested by synthetic lethality, consistent with the interpretation of genetic relationships uncovered by synthetic lethality that has emerged from extensive systematic genetic analysis in yeast [38]. These analyses have also been employed productively to elucidate the cytokinetic and cell separation processes in *S. cerevisiae* [39], [40], [17], [29], [20]. Amphiphysins have previously been implicated in aspects of cytokinesis in both *S. cerevisiae* and *S. pombe*. In budding yeast high level expression of actin resulted in the observation of a ring like actin structure at the bud neck that was physically and genetically distinct from the CAR, the formation of which was Rvs167-dependent [41]. In *S. pombe* Hob3, the Rvs161 orthologue, was shown to localize to the CAR in an actin dependent manner and in the null mutant the CAR contraction rate was reduced [42]. We therefore undertook to investigate a potential role for the budding yeast amphiphysin complex in cytokinesis in more detail and using a combination of genetic and imaging experiments demonstrate roles for the amphiphysin complex in CAR assembly and in the GEF independent accumulation of GTP bound Rho1 at the bud neck following CAR contraction.

## Results

### Synthetic lethality between *iqg1-1* and amphiphysin mutations

**Figure 1:**
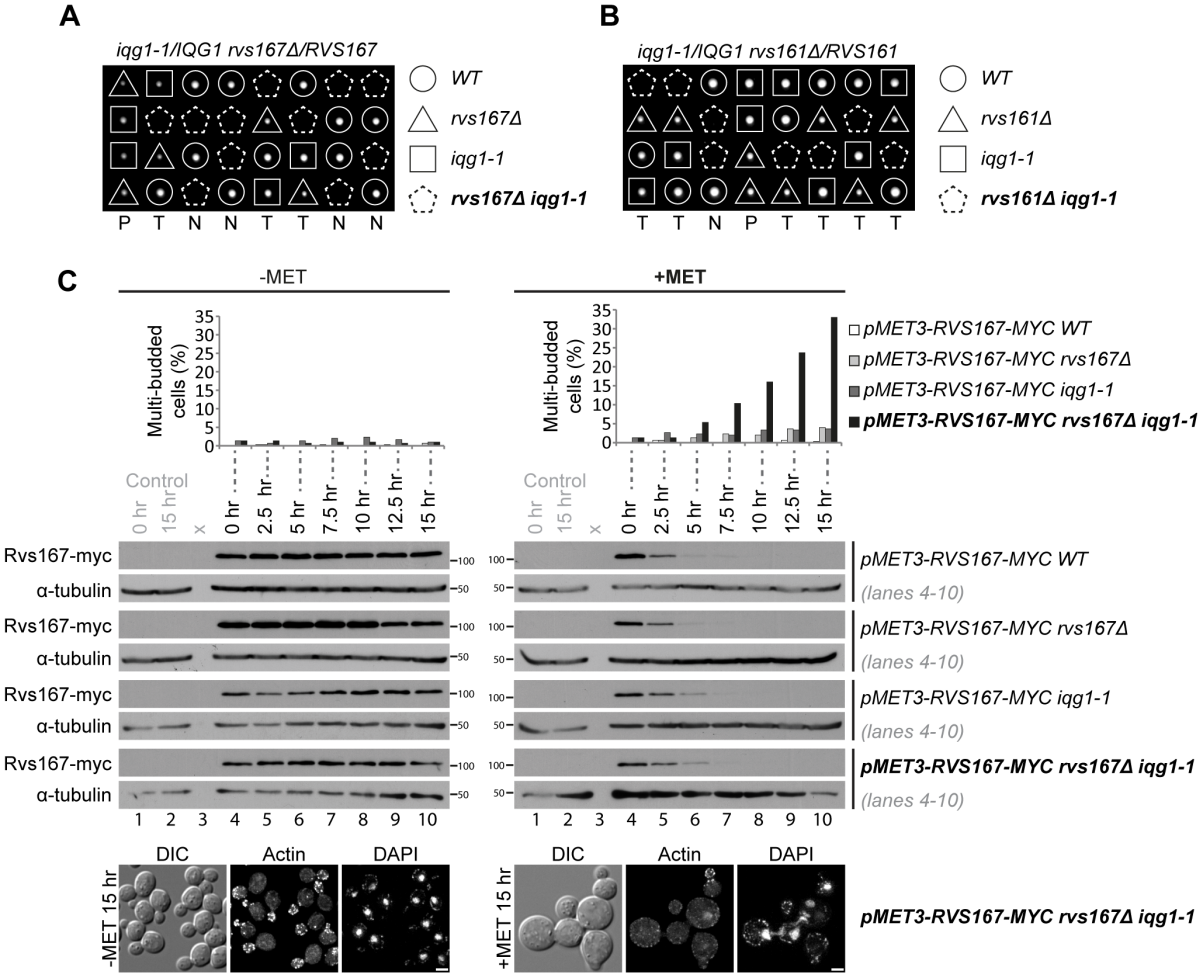
The null mutants *rvs167Δ* and *rvs161Δ* are synthetically lethal with the hypomorphic *iqg1-1* allele. Eight representative tetrads depicting (**A**) *rvs167Δ iqg1-1* synthetic lethality and (**B**) *rvs161Δ iqg1-1* synthetic lethality. The individual genotypes of the viable spores and the inferred double mutants are indicated, P = parental ditype, N = non-parental ditype, T = tetratype. **C** Repression of ectopic Rvs167-Myc expression recapitulates *rvs167*Δ *iqg1-1* synthetic lethality and the associated cytokinesis defect. Indicated strains were grown at 26°C, the permissive temperature for the *iqg1-1* allele, in media lacking (−MET) or containing (+MET) methionine and scored at the indicated time points for percentage of cells displaying multiple buds (n = 300 cells). This experiment was repeated twice but the data presented are representative and derived from a single experiment. Rvs167-13Myc protein levels were assayed by western blot following the addition of methionine and compared to a constitutive a-tubulin control. The two left hand control lanes in both panels represent wild type control levels of α-tubulin at the beginning and end of the time course. The micrographs depict representative examples of the actin and nuclear distribution in the *RVS167-13MYC rvs167Δ iqg1-1* cells after 15 hours growth −/+ methionine.

We initially screened a number of mutations in genes encoding proteins involved in the regulation and function of the actin cytoskeleton for genetic interaction with the hypomorphic, temperature sensitive *iqg1-1* allele. Three independent analyses all identified null mutations in *rvs161Δ* and *rvs167Δ* as being synthetically lethal in combination with *iqg1-1* at 26°C, the permissive temperature for the conditional allele. The results from standard genetic analysis of crosses between *iqg1-1* and *rvs167Δ* and *rvs161Δ* are presented in Figs. 1A and B respectively. In total 30 tetrads were analysed for *iqg1-1*×*rvs167Δ* yielding 5 parental ditypes, 6 non-parental ditypes and 19 tetratypes, close to the expected 1∶1∶4 ratio. In all cases [31] where co-segregation of the mutated loci was inferred spores were inviable on non-selective media at 26°C as indicated (Figs. 1A). In the *iqg1-1*×*rvs161Δ* cross dissection of 31 tetrads yielded 8 parental ditypes, 5 non-parental ditypes and 18 tetratypes and again in all cases of inferred co-segregation of the two mutations [28] cells were inviable (Fig. 1B). Control crosses to wild type for all three mutations demonstrated that none exhibited spore germination or viability problems (*iqg1-1* 93% viability and inviability did not segregate with the *iqg1-1* allele; *rvs167Δ* and *rvs161Δ* 100% viability). Microscopic examination revealed that all inviable double mutants exhibited a chained phenotpye and that lethality occurred after three or four attempts to complete cell division. In order to examine the terminal phenotype in more detail we created *iqg1-1* and control strains in which the sole source of the relevant amphiphysin gene was a 13-myc epitope tagged copy integrated into the genome under control of the *MET3* promoter, thus rendering amphiphysin gene expression methionine dependent. These strains were tested for their ability to restore normal amphiphysin function. *RVS167-13Myc* rescued all phenotypes associated with an *rvs167* null mutation; synthetic lethality with *iqg1-1* on methionine deficient media, salt sensitivity and actin cytoskeleton polarity (Fig. S1A, B and C). In contrast the *rvs161-13Myc* allele, despite being able to restore viability to *iqg1-1 rvs161Δ* cells (Fig. S1D), exhibited a degree of salt tolerance that was intermediate between wild type and *rvs161Δ* levels (Fig. S1E). As a result all subsequent depletion experiments described below focused on Rvs167. Initial experiments examined the morphological effects of Rvs167-13myc depletion in *iqg1-1* and control strains. The data show that, on depletion of the protein in *iqg1-1 rvs167Δ* cells, multiple budded, chained cell bodies with de-polarized actin accumulate over a 15 hr time course (Fig. 1C). The initial appearance of chained cells in the population at 7.5 hrs was coincident with loss of the protein as revealed by western blotting (Fig. 1C) and with the maxima of small to medium bud sized cells exhibiting depolarization of the actin cytoskeleton (Fig. S1C). It was concluded from these data that loss of amphiphysin function in a strain where Iqg1 function was compromised resulted in a failure of cytokinesis and cell separation.

### Cell cycle dependent Rvs167 localization to the bud neck

**Figure 2:**
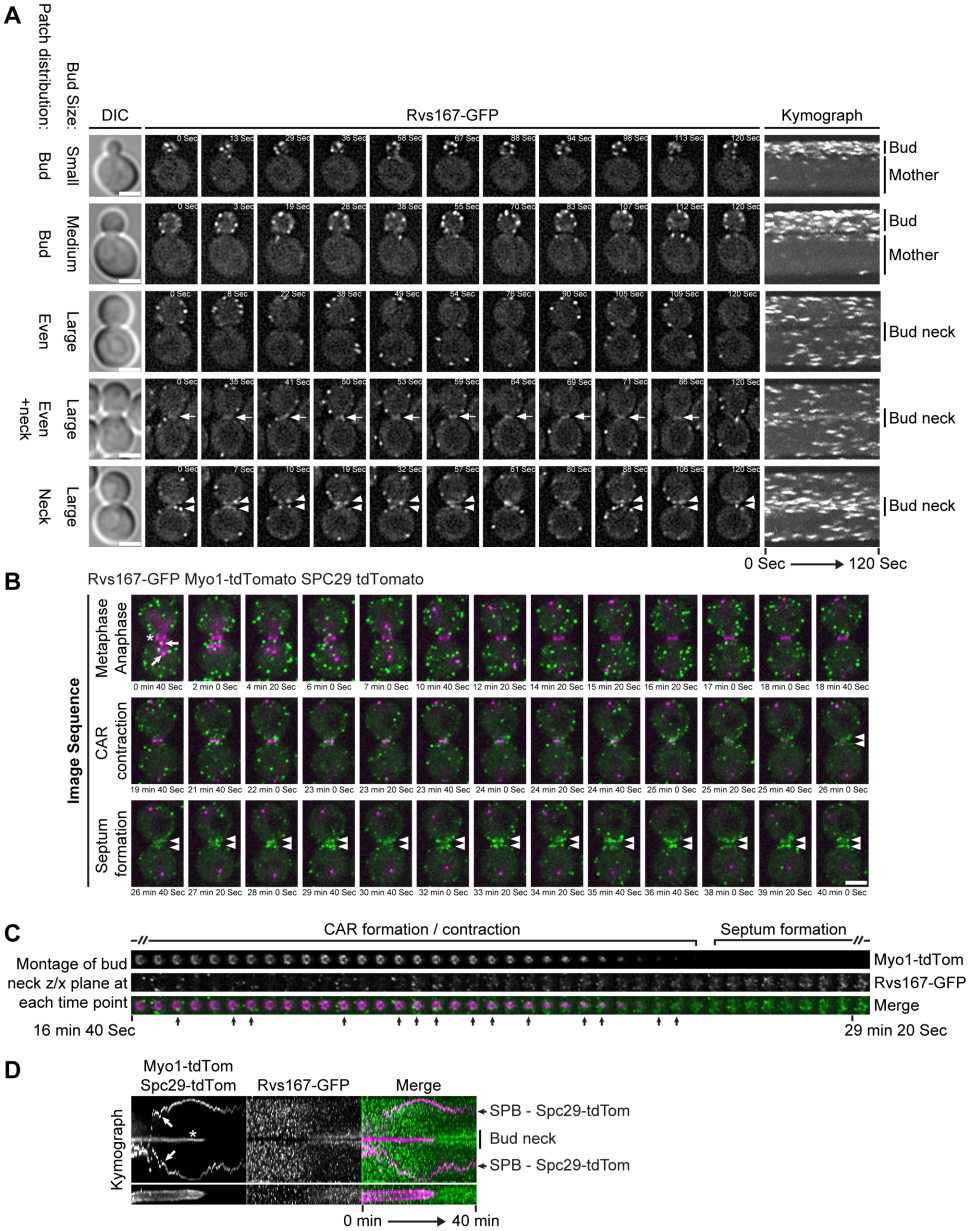
Cell cycle distribution of dynamic Rvs167-GFP in wild type cells. **A** representative single cell image series of Rvs167-GFP distribution at different stages of the cell cycle are shown. For each image series a single z-section that dissected the bud neck was imaged every 1 second for 2 minutes using 800 ms exposures. Images were deconvolved and are presented as stills or kymographs. Cell cycle stage was inferred from bud size and the protein distribution relative to bud size are summarized, DIC images correspond to last time point in the series. Arrows indicate transient Rvs167-GFP signal observed at the bud neck and arrowheads show polarization of the signal to either side of the bud neck in late mitosis. The right hand panels show the image series collapsed into kymographs and mother and daughter cells along with the position of the bud neck are labelled. Scale bars = 2.5 µm. (B–D) *GAL-CDC20 cdc20Δ RVS167-GFP MYO1*-*tdTomato SPC29*-*tdTomato* strain grown in minimal media containing galactose and raffinose and supplemented with excess adenine were shifted to minimal media containing glucose for 4 hrs 15 minutes to effect metaphase arrest. Cells were released from metaphase arrest by subsequent return to growth in galactose/raffinose containing minimal supplemented with excess adenine. The representative image series consist of 21z-sections (0.2 µm spacing) imaged every 20 seconds for 40 minutes. **B** the image series presented as maximum intensity projection stills from indicated time points. Arrow heads indicate Rvs17 bud neck localization, * indicates Myo1-tdTomato localization and arrows indicate Spc29-tdTomato fluorescence (i.e. position of the spindle pole body). **C** the same image series, as above, presented as a montage of z/x plane at each time point. **D** kymographs of same cell showing the Myo1-tdTomato and Spc29-tdTomato distributions depicted in magenta, indicated as in B, and the Rvs167 distribution in green.

If Rvs167 functions in cytokinesis/cell separation then it might be predicted that the protein localizes to the bud neck region and that potentially it interacts with known components of the cytokinetic and cell separation machinery. Using a fully functional Rvs167-GFP fusion expressed from the native locus the dynamics of Rvs167 distribution was examined. Representative images of Rvs167-GFP at different stages of the cell cycle are shown in Fig. 2A. The kymograph illustrates clear re-polarization of Rvs167-GFP patches to the bud neck in large budded cells forming either a transient ring like structure (Fig. 2A, arrows) or concentrating either side of division plane (Figure 2A, arrowheads). Next co-localization of Rvs167-GFP with Myo1-tdTomato relative to ring contraction was examined. In the time series depicted in Fig. 2B foci of Rvs167-GFP can be observed at the bud neck early in mitosis as the spindle pole bodies separate across the bud neck (40 sec–7 min) and at later stages coincident with the initiation of ring contraction (21 min 40 sec onwards). Thereafter there is a strong polarization of the signal following completion of ring contraction and CAR disassembly (26 min–40 min) as the spindle pole bodies transit from the periphery to the centre of the mother and daughter cells following mitotic spindle breakdown. An additional timed image reconstruction series through the alternative plane and focused entirely at the CAR again show that there are two phases of Rvs167 localization to the bud neck, the first coincides with the period immediately prior to and coincident with CAR contraction. During this phase highly dynamic small foci of fluorescence are observed and can be seen to co-localize with the CAR for relatively brief periods of time (Fig. 2C, arrows). The second phase of localization occurs immediately subsequent to the completion of CAR contraction, coincident with septum formation, and shows an increase in the number of Rvs167-GFP foci at the bud neck. To further refine the timing of Rvs167 recruitment to the bud neck region Rvs167-GFP localization was monitored in comparison with CAR contraction (Myo1-tdTomato) and spindle pole body movement (Spc29-tdTomato) and the data represented as merged kymographs (Fig. 2D). As indicated the data demonstrate that Rvs167 accumulates at the bud neck over a very short interval immediately prior to the initiation of CAR contraction and remains polarized to both mother and daughter sides of the bud neck for a substantial period of time thereafter (Fig. 2D). These two phases of Rvs167 localization suggested the possibility that the amphiphysin complex functions in both the CAR formation/contraction and septum formation pathways.

### Rvs167 interacts with known contractile ring and septation components

**Figure 3:**
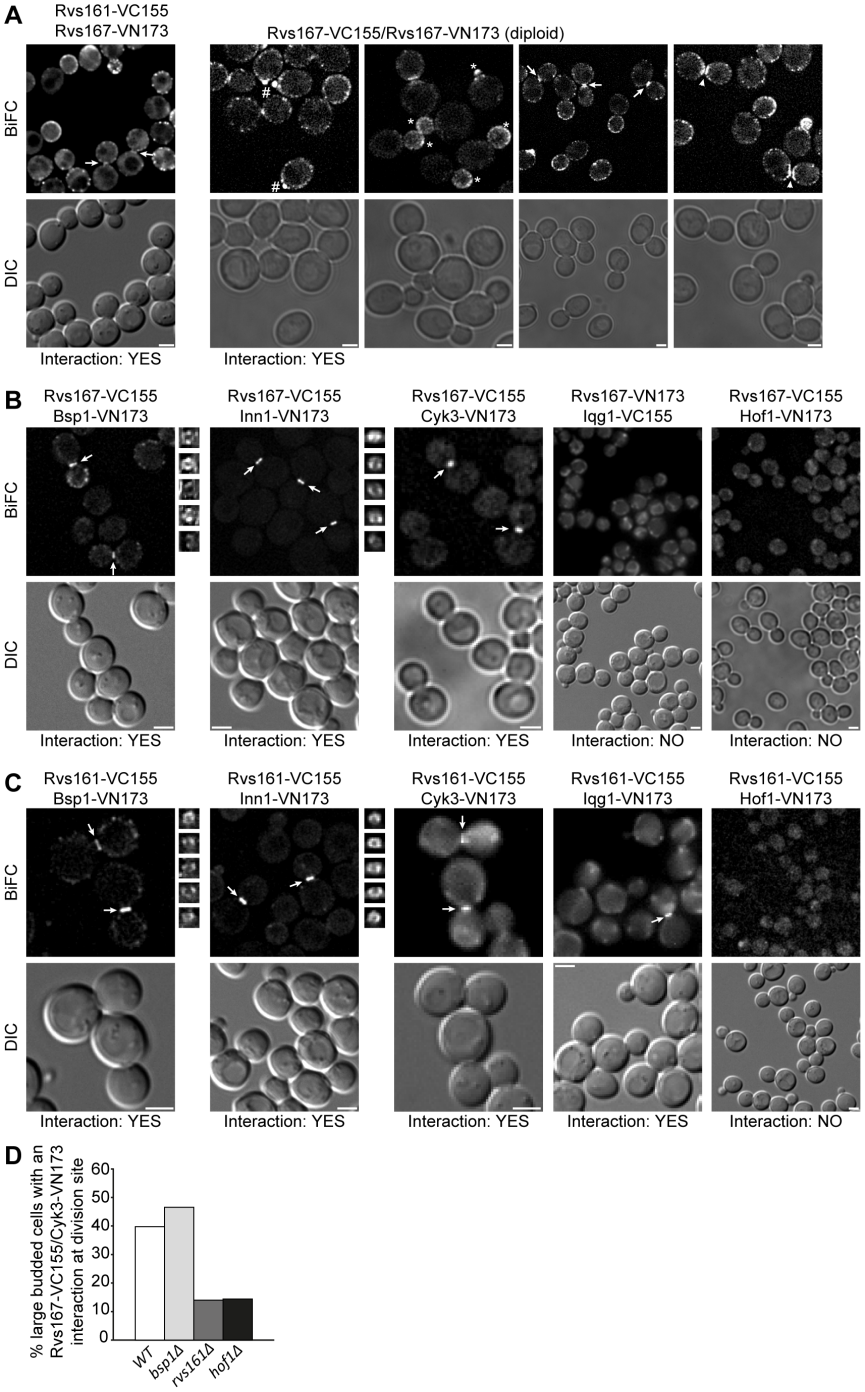
The amphiphysins interact with known contractile ring and bud neck components. In all panels live cell images of bimolecular fluorescence (BiFC) were acquired and are presented as projections of 21 z-sections each section spaced 0.2 um. **A** control BiFC images demonstrating interaction between Rvs161 and Rvs167, upper left panel, average intensity projection image. Maximum intensity projection image in the right hand panels demonstrate Rvs167 homodimerisation. The DIC images allow protein distribution to be assessed in relation to bud size. Arrows indicate apparent bud neck localization in large budded cells undergoing cytokinesis/cell separation; # indicates polarization of the signal at the incipient bud site and * in the bud of small budded cells. **B** Rvs167 displays BiFC interaction at the bud neck with Bsp1, Inn1 and Cyk3 as indicated by the arrows but does not interact Iqg1 or Hof1, all images are average intensity projections. **C** Rvs161 displays BiFC interaction at the bud neck with Bsp1, Inn1, Cyk3 and Iqg1 (average projection images), as indicated by the arrows but not Hof1 (maximum intensity projection). **D** Rvs167/Cyk3 BiFC interaction is dependent on Rvs161 and Hof1; WT (n = 176), *bsp1Δ* (n = 131), *rvs161Δ* (n = 157) and *hof1Δ* (n = 215). Pixel binning (2∶2) was used to detect the Rvs167/Cyk3, Rvs161/Cyk3 and Rvs161/Iqg1 BiFC signals. The n values derive from a single representative experiment.

As stated above if Rvs167 is involved in CAR assembly and co-localizes with the CAR then it might be anticipated that it interacts with CAR components. However from the data presented above (Figs. 2A and 2B) any such interaction is likely to be very transient. Bimolecular fluorescence imaging is a useful technique for the live imaging of protein/protein interactions and one property of the approach is that the interaction between the two components of the fluorophore dramatically stabilizes the interaction between the target proteins [43]. This property was exploited to examine the potential interactions between Rvs167 and other CAR components. As positive controls we tested for previously published BiFC interactions involving the amphiphysin proteins; namely potential Rvs167 homodimerization and Rvs167/Rvs161 heterodimerization observed through the oligomerization of the amphiphysin complexes [33]. Predictably Rvs167/Rvs167 and Rvs167/Rvs161 BiFC interactions were observed, occurring at sites of polarized cell growth, the bud, bud neck and was occasionally observed flanking the division site in large budded cells as indicated in Fig. 3A. As a further control interaction between Rvs167 and Bsp1 was tested. The data confirm this interaction, BiFC giving rise to patches at sites of polarized growth, consistent with the role of these two proteins in endocytosis, and as a ring structure at the CAR (Fig. 3B), of which Bsp1 is a known component [29]. However, because of the fact that BiFC stabilizes interactions it is possible that this interaction is established at patches and that Rvs167-VC155 is subsequently trafficked to the CAR as a passenger with Bsp1-VN173 and that the CAR localization seen in Fig. 3B does not reflect the normal behaviour of Rvs167. To address this possibility, interactions between Rvs167 and proteins associated with the CAR and primary septum formation, Iqg1, Cyk3, Inn1 and Hof1, which localize only to the bud neck were tested. Fluorescent signals at ring structures within the bud neck were observed for both Cyk3 and Inn1 but not with either Iqg1 or Hof1 (Fig. 3B). Interaction between Rvs161 and this group of proteins was also examined and Rvs161 localized to ring structures at the bud neck in association with Bsp1, Inn1, Cyk3 and additionally interaction with Iqg1 was observed, but again not with Hof1 (Fig. 3C). Because such well defined ring structures are not observed in cells expressing Rvs167-GFP we conclude that BiFC traps transient interactions between Rvs167 and its binding partners, Bsp1, Cyk3 and Inn1, at the bud neck. Interaction between Hof1 and Cyk3 has been well documented therefore the dependency of the Rvs167/Cyk3 interaction upon functional Hof1 was tested. The number of cells exhibiting a BiFC signal was significantly reduced in the absence of Hof1 function (Fig. 3D). Importantly the interaction was also reduced to similar levels in the absence of Rvs161 function indicating that the localization to the CAR is likely to be a property of the amphiphysin complex and not solely a property of Rvs167. In summary Rvs167 transiently co-localizes with Myo1 in early mitosis, prior to CAR contraction, and is able to interact with Cyk3 and Inn1 at the bud neck. In addition its obligate amphiphysin partner, Rvs161, is able to interact with Iqg1 a bone fide CAR component required for CAR assembly. Formally it remains possible that, despite the negative data reported above, Rvs167 also interacts with Iqg1 and Hof1 and that the relative positioning of the BiFC fluorophore domains in the respective fusion proteins prevents a positive fluorescent signal.

### CAR formation in mitosis is delayed in both *iqg1-1* and *rvs167* mutants

**Figure 4:**
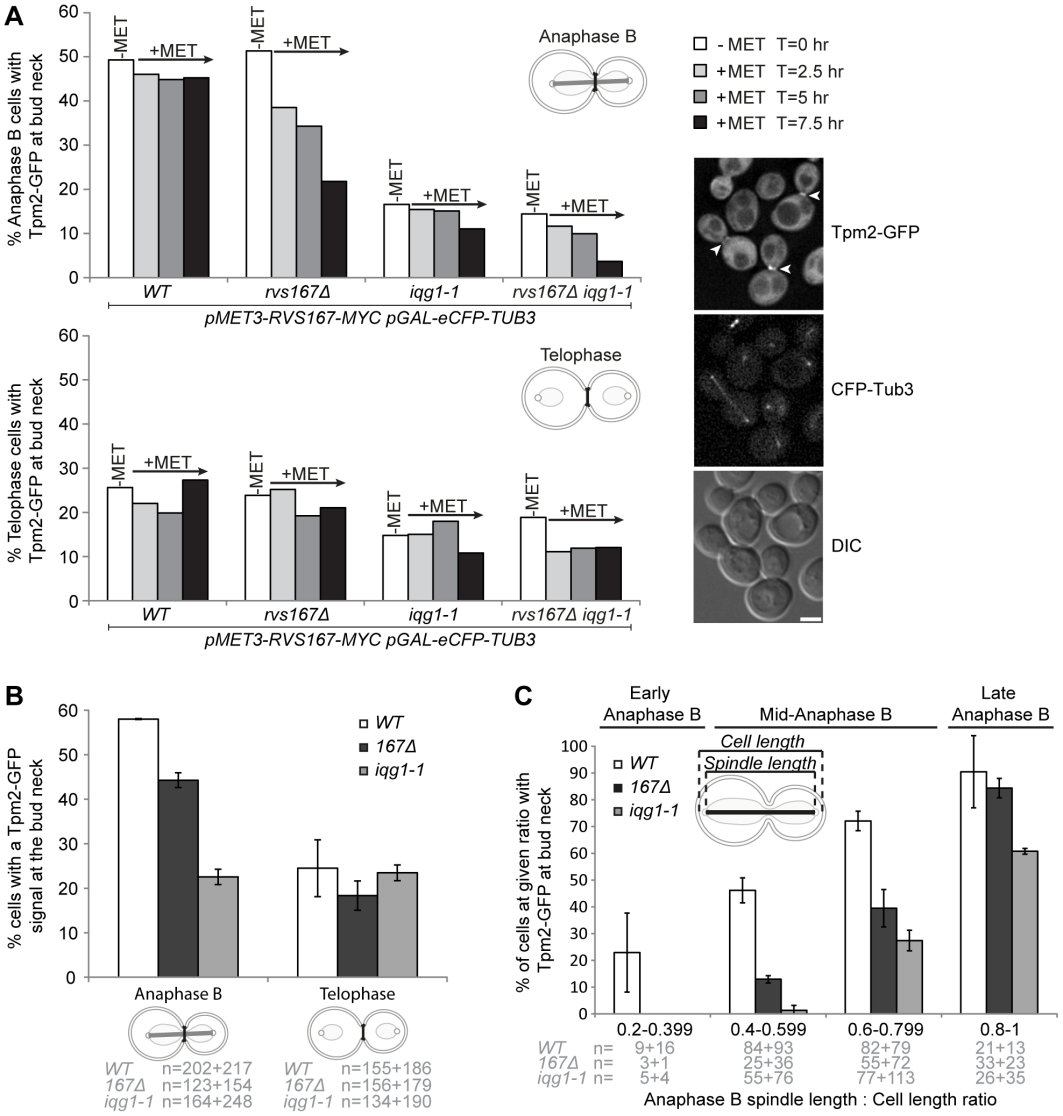
The timing of actin ring assembly is delayed in *iqg1-1* and *rvs167Δ* mutants. **A** indicated strains were grown to exponential phase in minimal media lacking methionine (−MET) before shifting to media containing methionine (+MET). Live cell images were acquired and the presence of Tpm2-GFP at the bud neck scored at the indicated time points. Mitotic progression stage was assessed by observation of mitotic spindle length using CFP-Tub3. Between 160–344 cells were scored from at each time point. The percent values derive from a single representative experiment. **B** Tpm2-GFP presence at the bud neck in wild type and single mutant parental strains. Cells were grown to exponential phase and live images captured and scored for Tpm2-GFP bud neck localization and anaphase B/post anaphase microtubule distribution (N values are indicated). **C** the same image set scored for Tpm2-GFP bud neck localization relative to mitotic progression. To quantify mitotic progression the ratio of anaphase spindle length to overall cell length was calculated and binned as indicated (N values as shown). The N values used in B and C derive from two independent repeats and the number of cells scored in each category in each experiment are indicated below the graphs.

As both Iqg1 and Rvs167 are involved in regulating aspects of actin cytoskeletal function the formation of the CAR was examined. To address this question the Rvs167-13Myc depletion strains described above were engineered to express a Tpm2-GFP fusion expressed from the native locus. *TPM2* encodes one of two *S. cerevisiae* tropomyosin orthologues and use of the GFP fusion enables live cell imaging of F-actin structures such as the CAR [24]. During anaphase B, the number of *iqg1-1* cells exhibiting Tpm2-GFP fluorescence at the bud neck was reduced relative to wild type indicating that even when grown at the permissive temperature, the *iqg1-1* allele is not fully functional. Importantly depletion of exogenous Rvs167-13Myc in cells lacking endogenous *RVS167* (*rvs167Δ*) caused severe impairment to CAR formation and this phenotype was exacerbated in the *iqg1-1 rvs167Δ* double mutant (Figure 4A). In contrast, in telophase cells (marked by spindle break down), both wild type and Rvs167 depleted cells shared similar numbers of CAR containing cells whilst in the *iqg1-1 rvs167* double mutant CAR numbers increased post-anaphase only to the parental *iqg1-1* level. Rvs167-13Myc depletion had little effect on CAR formation at this cell cycle stage (Fig. 4A). These data suggest that CAR formation is biphasic with both Iqg1 and Rvs167 functioning to promote CAR formation in early anaphase B. Moving away from the set of depletion strains described above, we also examined CAR formation in wild type, *iqg1-1* and *rvs167Δ* cells. Again we observed a reduction in anaphase B CAR formation in both mutant strains relative to wild type and CAR assembly appeared to be restored in telophase cells. To examine the anaphase B defect in more detail anaphase B cells were further sub-divided on the basis of mitotic spindle length to cell length ratio. Spindle length in anaphase B cells was divided by the overall cell length, as determined by the boundaries of cytoplasmic Tpm2-GFP fluorescence. Broadly the data demonstrate the same trend as above, namely that CAR formation is compromised in *iqg1-1* and *rvs167Δ* cells during early anaphase B and CAR formation is restored late in the mitotic cycle, once cells fall within a class in which the ratio of spindle length to overall cell length lies between 0.8 and 1.0 (Fig. 4B). It is important to note that in this experiment there is 2.65 fold increase, relative to wild type, in the number of *rvs167Δ* anaphase B cells that fall within this latter classification (0.8–1.0). This means that if one simply amalgamates early, middle and late anaphase B stages into one group, the severity of the CAR formation defect associated with *rvs167Δ* cells is somewhat hidden (Fig. 4B). These data are consistent with the interpretation that both Iqg1 and Rvs167 play a role in CAR assembly during anaphase B and that subsequently a second mechanism dependent upon factors other than Rvs167 either assembles or stabilizes the CAR in late anaphase B and post-anaphase.

### Synthetic lethality is independent of CAR assembly defects

**Figure 5:**
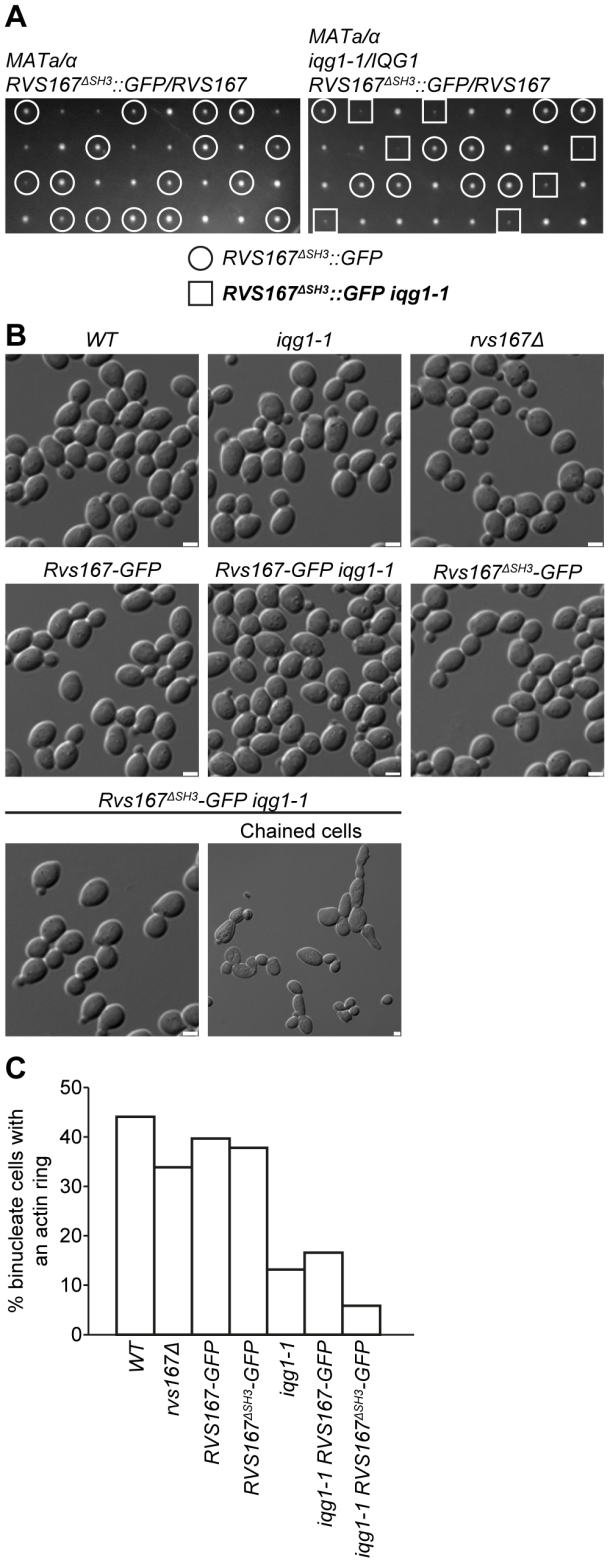
The SH3 domain of Rvs167 is required for actin ring assembly. **A** representative tetrad analysis of the relevant heterozygous diploid strains and the relevant segregation pattern of the *rvs167^ΔSH3^-GFP* and *iqg1-1* alleles. **B** the indicated strains were grown to exponential phase in YEPD at permissive 26C before fixation (3.7% formaldehyde) and DIC image acquisition. Scale bars = 2.5 µm. **C** formaldehyde fixed cells from above were stained with tritc-phalloidin (actin) and DAPI (DNA). WT (n = 295), 167 d (n = 304), *RVS167-GFP* (n = 290), *RVS167^ΔSH3^-GFP* (n = 344), *iqg1-1* (n = 243), *iqg1-1 RVS167-GFP* (n = 452), *iqg1-1 RVS167^ΔSH3^-GFP* (n = 222). The n values derive from a single representative experiment.

The question remained as to whether the failure of CAR assembly correlated to loss of cell viability in *rvs167Δ iqg1-1* and *rvs161Δ iqg1-1* cells. To address this question we created strains in which the SH3 domain of Rvs167 was deleted and replaced by insertion of GFP. Genetic analysis revealed that double mutants between *iqg1-1* and *rvs167ΔSH3::GFP* were viable (Fig. 5A). In total 47 tetrads were analyzed and 47/48 double mutants were viable (98%) however they had a reduced growth rate relative to WT and single mutant progeny (Fig. 5A) coupled to a chained morphological phenotype as observed by microscopic examination (Fig. 5B). Subsequently cells were assessed for CAR formation and the results demonstrate that *rvs167ΔSH3::GFP* cells exhibit only a very mild reduction in CAR formation compared to controls. However in combination with *iqg1-1* the *rvs167ΔSH3::GFP* allele caused a severe reduction in CAR formation further compromising the reduced levels observed in the *iqg1-1* single mutant. These data implicate the SH3 domain of Rvs167 in CAR formation but imply that additional amphiphysin function is required to maintain viability in *iqg1-1* cells. Consistent with this interpretation was the observation that simultaneous deletion of the GPA and SH3 domains of Rvs167 further reduced spore viability of double mutants with *iqg1-1* (data not shown) and a Rvs167 BAR-GPA-SH3 domain deletion resulted in synthetic lethality (Fig. 1A).

### CAR contraction is compromised in *iqg1-1* at the permissive temperature but retains normal contractile behaviour in the absence of Rvs167 function

**Figure 6:**
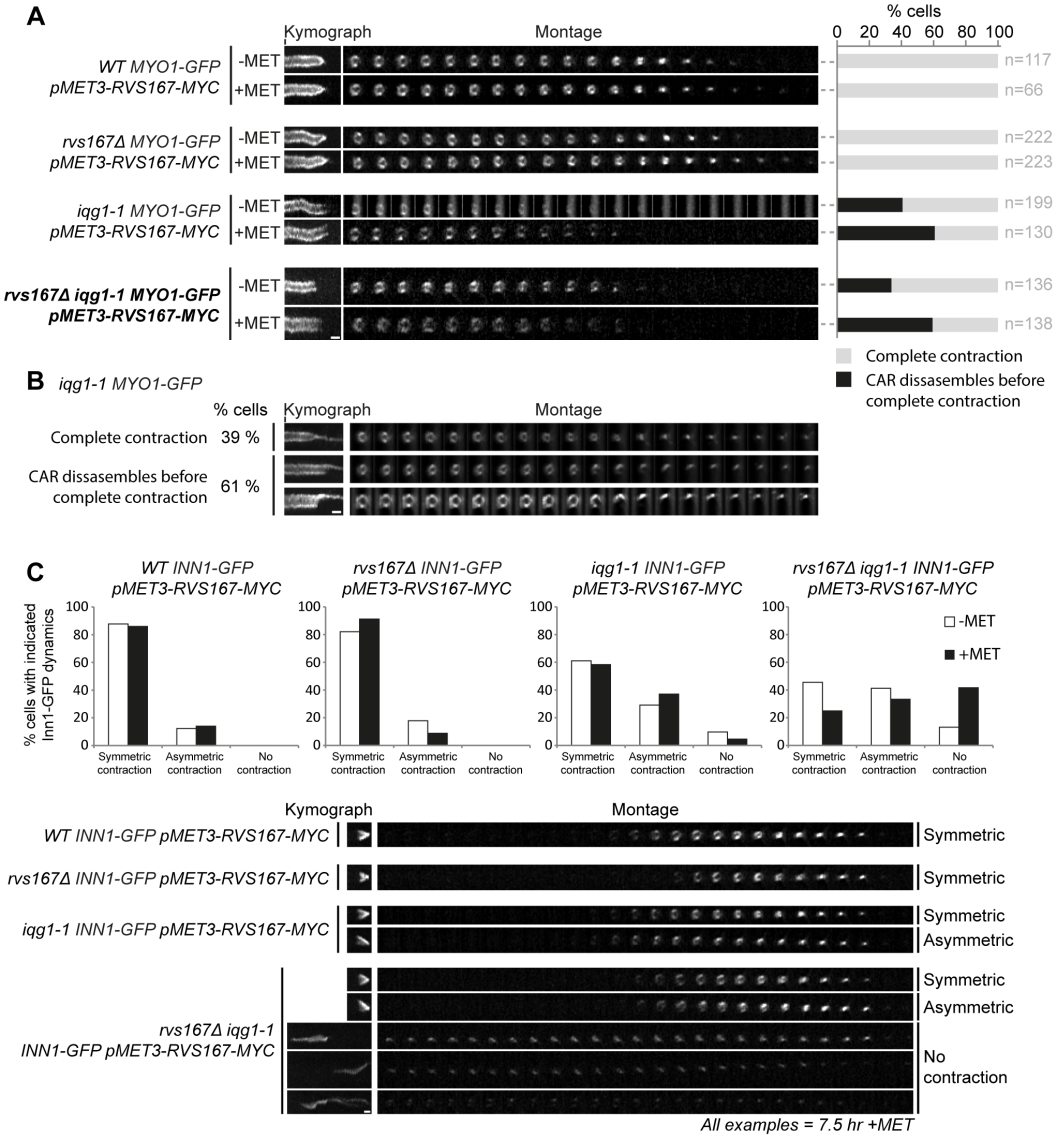
Actomyosin contractile ring and Inn1-GFP dynamics are altered in *iqg1-1* and *iqg1-1 rvs167Δ* mutants. **A** the indicated strains were grown to exponential phase in media lacking methionine and then imaged (−MET). Exponential −MET cells were transferred to media containing methionine and grown for 7.5 h to deplete Rvs167-13myc before imaging (+MET). Images were acquired every 35 seconds for 50 minutes (21z-sections, 0.2 µm spacing). Kymograph scale bar = 3.5 mins. **B** CAR contraction fails in *iqg1-1* cells. Images acquired every 45 seconds for 45 minutes (21z-sections, 0.2 µm spacing). Kymograph scale bar = 6 mins. **C** the indicated strains were grown and imaged as described above. Kymograph scale bar = 3.5 mins. Cell numbers scored for symmetric, asymmetric or no contraction were: WT (n = 138), *rvs167Δ* (n = 145), *iqg1-1* (n = 113), *rvs167Δ iqg1-1* (n = 274). The n values in A and C derive from a single representative experiment.

Next the contractile function of the CAR was examined in the strains indicated using live cell imaging of a Myo1-GFP fusion to monitor contraction. Examination of the representative image time series and the kymographs constructed from those series demonstrate that CAR contraction goes to completion in wild type and Rvs167 depleted cells (Fig. 6A). However all strains carrying the *iqg1-1* allele are severely compromised for CAR contraction. The kymographs fail to taper to the point of complete contraction and Myo1-GFP marked CAR imaged in the alternative plane, observed through the bud neck, can be seen to disassemble without contracting (Fig. 6A). These observations were confirmed in asynchronously growing *iqg1-1 RVS167 MYO1-GFP* cells where CAR contraction failed and rings disassembled in 61% cells (Fig. 6B). In those cells where potential contraction was observed it often appeared asymmetric which might indicate that apparent contraction was an observational artefact resulting from collapsed CARs.

Primary septum formation is coupled to CAR contraction and can be represented by the dynamics of Inn1 behaviour that follows the progress of membrane ingression and is required for Chs2 activation [44]. Inn1-GFP contraction/ingression was seen to occur similarly in wild type and *rvs167Δ* cells (Fig. 6C). However in *iqg1-1* cells an increase in aberrant Inn-GFP movement was observed and in a small proportion of cells no contraction occurred. Depletion of Rvs167-13myc in the *iqg1-1 rvs167Δ* background increased the number of cells in which Inn1 ingression was absent. These data indicate that Rvs167 has no role in CAR contraction but may play a role in co-ordinating the CAR and the proteins required for primary septum formation. The data further implicate Iqg1 in contraction as well as assembly of the CAR.

### Endocytosis required for CAR independent suppression of *iqg1-1* at the restrictive temperature but not for *iqg1-1* viability

**Figure 7:**
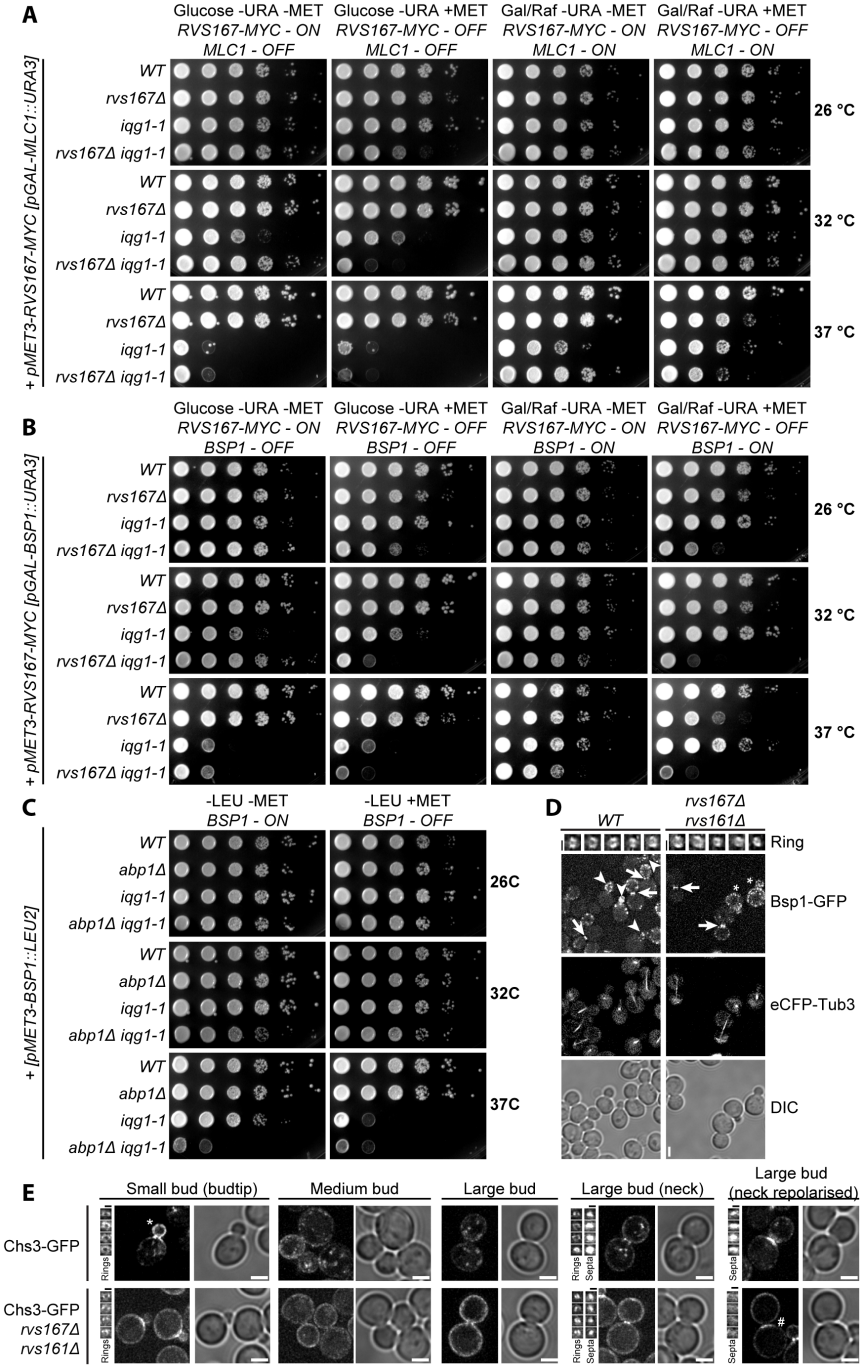
MLC1 overexpression, but not BSP1, suppresses *rvs167Δ iqg1-1* synthetic lethality. Indicated strains were grown to exponential phase in glucose containing media, washed into galatose/raffinose media and then spotted onto indicated media and grown at temperature shown before imaging. MLC1 overexpression suppresses *rvs167Δ iqg1-1* synthetic lethality (**A**) whereas BSP1 overexpression does not (**B**). Endocytosis is required for BSP1-mediated suppression of *iqg1-1* at the restrictive temperature (**C**). **D** BSP1 localisation unaltered in amphiphysin null cells. BSP1-GFP and eCFP-TUB3 represent maximum and average intensity projections respectively. 5 representative examples of BSP1-GFP rings at the bud neck in the z/x plane (i. e. looking through the division site) are shown. Scale bar = 2.5 µm. (E) Chs3-GFP polarity altered in *rvs* mutant cells. Fluorescent images are maximum intensity projections. Image reconstructions of bud neck demonstrate Chs3-GFP localizes to rings and septa. Scale bar = 2.5 µm.

The fact that CAR assembly and contraction defects failed to explain the observed synthetic lethality between *iqg1-1* and amphiphysin null mutations lead us to investigate other known aspects of amphiphysin function. Previous work has identified two dosage suppressors of the *iqg1-1* allele that define two distinct suppression pathways. High dosage expression of Mlc1 restores CAR formation to *iqg1-1* cells at the restrictive temperature whereas increased expression of Bsp1 leads to CAR independent cell separation through a default pathway requiring extensive but disorganized deposition of septal/cell wall components [16], [17]. The data presented in Fig. 7A demonstrate that increased Mlc1 expression is able to restore substantive growth to *iqg1-1* cells in the absence of Rvs167 expression. In contrast increased Bsp1 expression fails to restore viability to *iqg1-1* cells lacking Rvs167 expression (Fig. 7B). One possibility that emerges from these data is that it is the endocytic function of Rvs167 that is required both for viability of *iqg1-1* cells and for Bsp1 mediated suppression. Loss of endocytic function is unlikely to explain the observed synthetic lethality as double mutants between *iqg1-1* and *abp1Δ* retain viability (Fig. 7C) as do *iqg1-1 sla1Δ* and *iqg1-1 crn1Δ* cells (unpublished data). However CAR independent *iqg1-1* suppression does require efficient endocytosis as increased Bsp1 expression fails to suppress loss of *iqg1-1* function in *iqg1-1 abp1Δ* cells at the restrictive temperature (Fig. 7C). The failure of Bsp1 mediated dosage suppression of *iqg1-1* in the absence of Rvs167/Rvs161 does not result from altered localization of Bsp1 to the CAR (Fig. 7D). Actin patch localization of Bsp1-GFP exhibits the polarity defect previously associated with amphiphysin mutations [34].

Previous data demonstrated that Chs3 is required for *BSP1* dose dependent suppression of *iqg1-1* (29) suggesting the possibility that Rvs167 might regulate Chs3 localization/activity. We therefore examined Chs3-GFP localization in the absence of amphiphysin function. It had previously been shown that endocytic mutants exhibit increased levels of chitin synthase activity reflected in an accumulation of Chs3 throughout the plasma membrane as opposed to a polar distribution within the bud and a loss of chitosomes, both phenotypes resulting from a defect in recycling of Chs3 at the plasma membrane [46]. Chs3 localization in *rvs167Δ rvs161Δ* cells is consistent with those earlier reports i. e. the absence of chitosomes and altered plasma membrane distribution (Fig. 7E). Chs3 still accumulated at the plasma membrane surrounding the bud neck and marked closed septa in large budded cells but the level of the protein at the bud neck appeared to be markedly reduced in amphiphysin mutants compared to control cells (Fig. 7E).

A further possibility is that machinery required for primary septum formation is altered in amphiphysin deficient cells, although it should be noted that neither *rvs167Δ* nor *rvs161Δ* cells exhibit known septation defects. We were unable to test Chs2 localization as epitope tagged alleles of this gene are inviable in our strain background. We did examine the behaviour of functional Hof1-GFP and Cyk3-GFP, thought to be required for Chs2 activation [44] in *rvs167Δ* cells and found both proteins to undergo similar dynamic localization patterns as observed in wild-type cells (Fig. S2). One important implication of this result is that there is no obvious affect on general bud neck structure in the absence of Rvs167, consistent with normal progress of Inn1-GFP ingression, which tracks primary septum formation (Fig. 6C).

### Rom2 distribution is altered in *rvs167Δ* mutant

**Figure 8:**
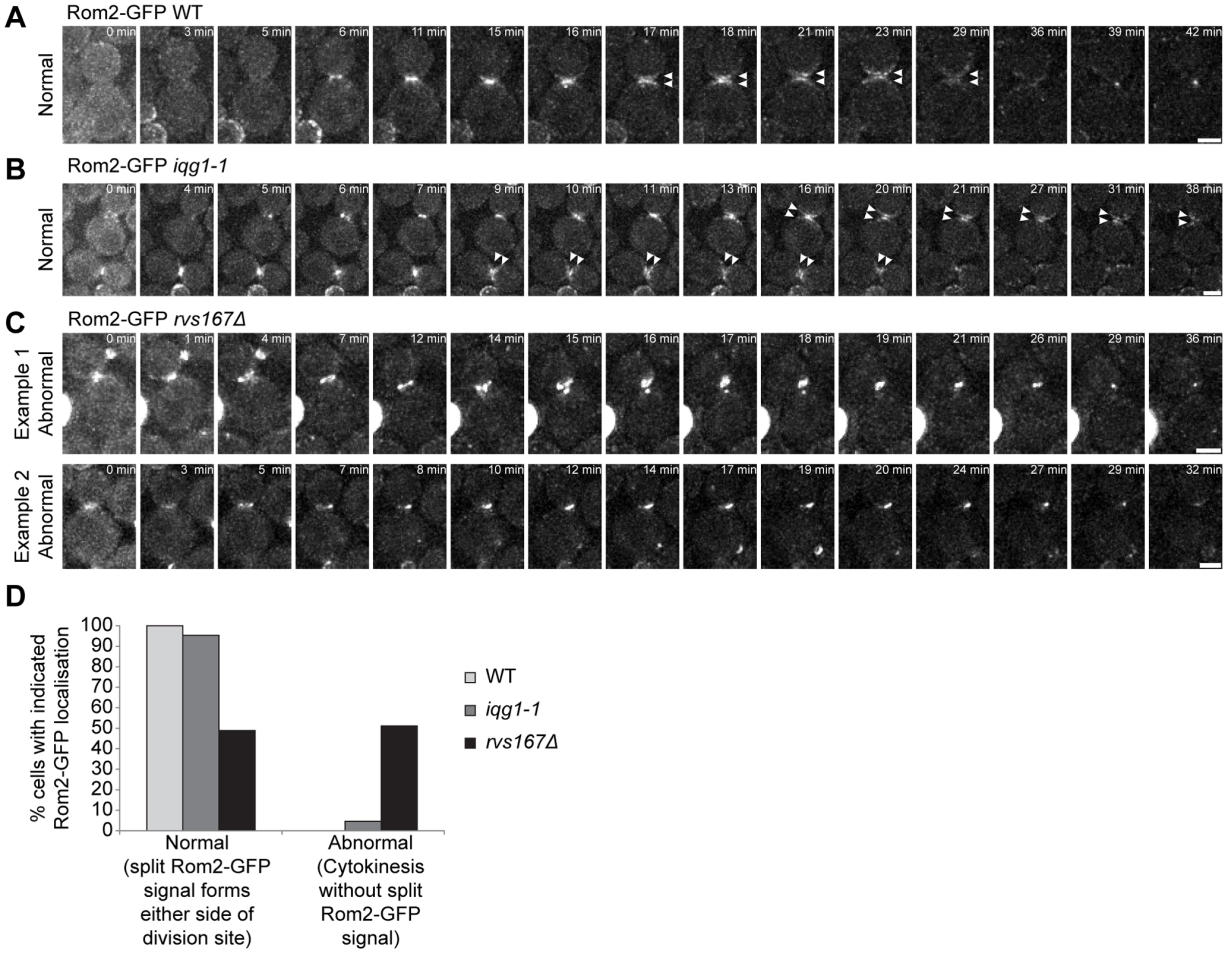
Rom2-GFP fails to repolarise at the division site prior to cell separation in *rvs167Δ* cells. WT (**A**), *iqg1-1* (**B**) and *rvs167Δ* (**C**) cells expressing Rom2-GFP were imaged every minute (3 second exposures, 18 z-sections, 0.2 µm z-spacing) for 45 minutes. Maximum intensity projections of deconvolved z-stacks are shown for indicated time points. Scale bars = 2 µm. (**D**) Percentage of WT (n = 54), *iqg1-1* (n = 44) and *rvs167Δ* (n = 42) cells that repolarized Rom2-GFP to either side of the bud neck prior to cell separation was quantified for each strain. The n values derive from a single representative experiment.

The altered distribution of Chs3 suggested the possibility that post-mitotic polarity establishment associated with secondary septum deposition and cell wall biosynthesis might be altered in *rvs167Δ* cells. The one other phenotype associated with a lack of amphiphysin function is the perturbation of actin cytoskeletal polarity [34]. The data presented in Fig. 8 demonstrate that dynamic behaviour of Rom2, one of two GEFs for Rho1, is altered in cells lacking amphiphysin function. In wild type and *iqg1-1* cells Rom2-GFP accumulates at the bud neck in late mitosis and then distributes evenly to either side of the bud neck in parallel with secondary septum formation (Fig. 8A and B). In the absence of Rvs167 function Rom2 fails to accumulate evenly on either side of the bud neck (Fig. 8C). The second known Rho1-GEF, Tus1, behaves normally in all three strains (Fig. S3).

### Rvs167 is required for Rho1-GTP localization to the bud neck

If normal Rom2 distribution is required for Rho1 activation at the point of cell separation then absence of Rvs167 might perturb Rho1 activity. Rho1 is known to function in polarity establishment during cytokinesis and cell separation and is required for secondary septum formation [25], [26]. Rho1 undergoes two phases of localization, one GEF dependent requiring GDP to GTP bound Rho1 cycling during late mitosis and the second GEF independent accumulation of the GTP bound form requiring the poly-basic Rho1 C-terminus and likely interaction with PIP2 [25]. Using GFP-Rho1 and the GFP-Rho1-Q68H mutant [25], a GTP-locked form of Rho1, we examined the GEF independent localization of Rho1 in the presence and absence of Rvs167. Here we observed a strongly altered distribution pattern with a loss of polarization of GFP-Rho1-Q68H to the bud neck in telophase cells and an even distribution around the plasma membrane of both mother cell and buds in sharp contrast to wild type cells (Fig. 8).

## Discussion

Amphiphysins are a conserved family of eukaryotic proteins involved in clathrin mediated endocytosis [33]. The budding yeast amphiphysin complex is a heterodimer formed between Rvs161 and Rvs167 with additional functions in salt tolerance and actin cytoskeletal polarity establishment [45]. Genetic analysis has demonstrated that *rvs161* and *rvs167* null mutations exhibit identical interactions which coupled to characterization of heterodimer formation and protein-protein interactions has led to the conclusion that the two proteins function as an obligate heterodimer [47]. Previous work has identified at least one phenotypic difference between the two mutants [48]. However, apart from the distinct BiFC interaction between Rvs161 and Iqg1 (Fig. 3C) that we interpret to reflect a genuine interaction of the Rvs161/Rvs167 complex, we have never observed a phenotypic difference between the *rvs161* and *rvs167* null mutations in the course of these experiments. Here we have shown that *rvs161* and *rvs167* null mutants exhibit synthetic lethality with the hypomorphic *iqg1-1* allele (Fig. 1). Iqg1, the sole *S. cerevisiae* IQGAP homologue, functions during cytokinesis and cell separation, it is a component of the CAR and is required for actomyosin ring assembly. Synthetic lethality between null and hypomorphic mutations implies that the proteins function in the same pathway [38] and we therefore conclude that the Rvs161/Rvs167 apmhiphysin complex functions in cytokinesis and cell separation. Consistent with this interpretation depletion of either Rvs161 or Rvs167 in an *iqg1-1* strain resulted in the appearance of chained cells (Fig. 1C). Analysis of the temporal and spatial pattern of an Rvs167-GFP fusion protein reveals transitory interactions with CAR reflected by co-localization with Myo1 (Fig. 2C). These interactions are observed prior to the initiation of CAR contraction (Fig. 2C and D). As contraction occurs Rvs167-GFP begins to accumulate further at the bud neck and persists beyond the point of CAR disassembly and completion of the cytokinetic phase of cell division, (Fig. 2A, B and D), behaviour that parallels the well characterized re-polarization of the actin cytoskeleton at this stage of the cell cycle.

On the basis of the results discussed above, that Iqg1 and the amphiphysins function in the same pathway(s) and Rvs167 transiently associates with the CAR a potential role for Rvs167 in CAR assembly was examined. In wild type a significant proportion (50%) of anaphase B cells have completed the assembly of the CAR. Depletion of Rvs167 reduces this level and *iqg1-1* cells also exhibit a reduction in the number of CAR containing anaphase B cells. This defect is exacerbated in double *rvs167Δ iqg1-1* mutants (Fig. 4A). However in post-anaphase cells CAR numbers are reduced in *iqg1-1* cells but Rvs167 depleted cells attain similar levels to wild-type cells (Fig. 4A). Further analysis demonstrated that in *rvs167Δ* and *iqg1-1* mutants CAR assembly is compromised during the early stages of anaphase but that cells are able to recover this defect and assemble wild type (*rvs167Δ*) or near wild-type (*iqg1-1*) CAR numbers. These data imply that an additional factor can license CAR assembly in late mitosis and one candidate for this is Hof1p as a *hof1* null mutant is synthetically lethal with *iqg1-1* [49] and *rvs167Δ* [50].

Rvs167 domain structure has been extensively studied and most functional aspects of the protein ascribed to the BAR domain which is associated with membrane binding and curvature sensing/induction [51], [37]. Construction of different domain deletion constructs at the endogenous locus and analysis of the genetic interaction between these mutations and *iqg1-1* revealed several important observations. First and unsurprisingly double mutants between *iqg1-1* and an Rvs167 construct lacking all three defined domains (BAR, GPA and SH3) exhibited synthetic lethality. Double mutants with a construct deleted for the GPA and SH3 domains, therefore retaining only the BAR domain, exhibited significantly reduced viability (data not shown). Double mutants which expressed Rvs167 lacking only the SH3 domain were viable although slow growing (Fig. 5B). Importantly though the double mutants showed a severe reduction in the number of bi-nucleate cells with observable CAR and morphologically exhibited high levels of chaining. The fact that these cells were viable yet the *rvs167* null and BAR domain construct were synthetically lethal with *iqg1-1* demonstrates whilst loss of CAR function compromises cytokinesis and cell separation it is not responsible for the observed loss of viability. Rather it is the loss of some other aspect of amphiphysin function that leads to cell death in combination with the *iqg1-1* mutation. Previous studies have identified a number of interacting partners for the Rvs167 SH3 domain and functionality when endocytosis is compromised [52]. The data presented here demonstrate that the SH3 domain of Rvs167 also functions in CAR assembly and are consistent with recent data demonstrating redundancy between the Rvs167 and Hof1 SH3 domains in actin ring assembly [50].

In order to further understand the function(s) of the amphiphysin complex during cytokinesis and cell separation we examined interaction between Rvs161, Rvs167 and components of the CAR (Iqg1 and Bsp1) and the chitin synthase 2 activation complex, Inn1, Hof1 and Cyk3. This second complex forms at the bud neck in late mitosis and is governed by the activity of the mitotic exit network [20]. Ingression of the plasma membrane and construction of the primary septum by Chs2 are guided by CAR contraction. Bi-molecular fluorescence clearly demonstrates interaction of both components of the amphiphysin complex with Inn1 and Cyk3 at the division site. The interaction of Rvs167 with Cyk3 is dependent upon Hof1 and Rvs161 indicating that these interactions represent genuine complex formation. Assembly of this complex has to be co-ordinated with CAR assembly and contraction and Rvs161, but not Rvs167, was found to interact with Iqg1. This interaction may reflect a role for the amphiphysin complex in Iqg1 recruitment as Iqg1 CAR localization is abolished in *rvs167 hof1* double mutants [50]. One interpretation of these data is that the amphiphysin complex acts in CAR assembly and physically bridges between the CAR and Chs2 activation complex thus acting to co-ordinate the two activities. We therefore examined the contractile behaviour of the CAR and the dynamics of Inn1 in *iqg1-1*, *rvs167*Δ and *iqg1-1 rvs167*Δ mutants. The presence or absence of Rvs167 alone had no influence on CAR contraction (Fig. 6A). However in *iqg1-1* cells CAR contraction was severely compromised and the CAR simply disassembled, often asymmetrically (Fig. 6A and B). Depletion of Rvs167 further compromised CAR contraction (Fig. 6A). Similarly no difference between wild type and *rvs167*Δ cells was observed when Inn1-GFP ingression was followed. However in *iqg1-1* cells Inn1 ingression was seen to follow an asymmetric path in a significant number of cells and in the *iqg1-1 rvs167* double mutant the majority of cells exhibited either asymmetric Inn1-GFP ingression or ingression failed entirely. Taken together with the protein-protein interactions observed between Iqg1, Inn1 and the amphiphysin complex these data raise the possibility that in addition to a role in CAR assembly the yeast amphiphysin complex co-ordinates CAR and Chs2p activation complex functions.

The fact that CAR contraction fails in *iqg1-1* is intriguing in the light of recent evidence demonstrating that the force generation required for contraction derives from actin disassembly and that Myo1 serves as a CAR scaffold but is not required for contractile force [53], [54]. Iqg1p was suggested to act as a cross-linker between actin filaments [55], a necessary function if contractile force is driven by filament disassembly, and the data presented here are consistent with that proposal.

As stated above amphiphysin function in CAR assembly and furrow ingression cannot explain the observed synthetic lethality we therefore examined other previously described aspects of amphiphysin function in yeast. We first tested whether endocytosis was required for *iqg1-1* cells to maintain viability. The data demonstrated that efficient endocytosis was unlikely to be required for viability but was necessary for CAR independent, Bsp1 mediated, suppression of the *iqg1-1* allele. This requirement presumably reflects the loss of vesicle recycling in the absence of endocytosis as evidenced by altered Chs3 distribution.

**Figure 9:**
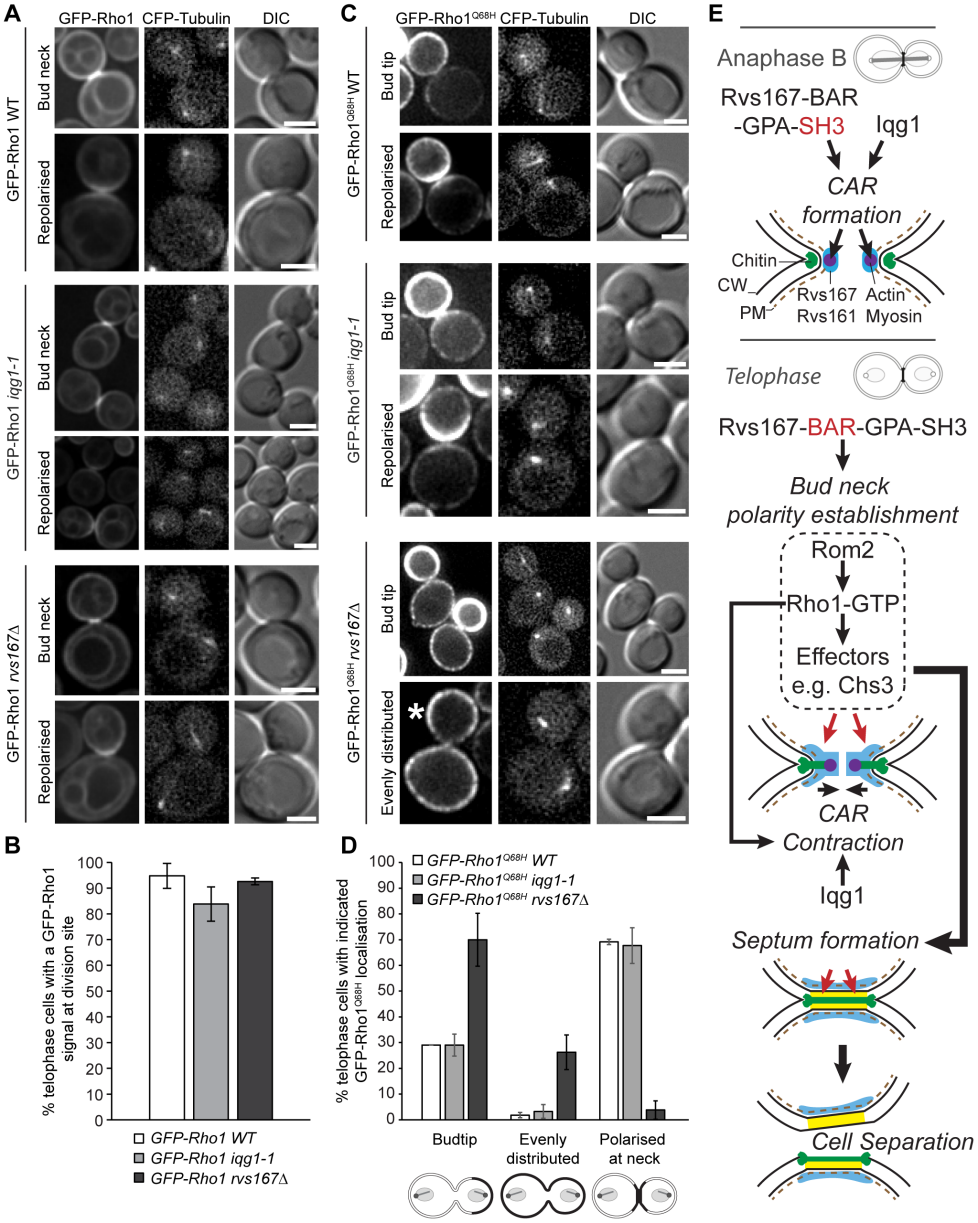
Rvs167 is required to localise active Rho1-GTP to the division site. **A** localisation of a wild-type GFP-Rho1 construct to the bud neck in telophase cells was largely unaltered in *iqg1-1* and *rvs167Δ* mutants as shown in the fluorescent average intensity project images. Scale bar = 2.5 µm. **B** Quantitation of GFP-Rho1 localisation to the division site in telophase cells (n = 2). In total 169, 163 and 211 telophase WT, *iqg1-1* and *rvs167Δ* cells were scored between two independent experiments and the scale bars represent standard deviation. **C** GTP-locked GFP-Rho1^Q68H^ mutant fails to repolarize from bud tip to bud neck upon spindle breakdown in *rvs167Δ* mutants, instead it often exhibited depolarized plasma membrane localisation (asterisk). **D** quantitation of GFP-Rho1^Q68H^ telophase localization (n = 2). In total 289, 301 and 221 telophase WT, *iqg1-1* and *rvs167Δ* cells were scored between two independent experiments. Error bars in B and D are +/− standard deviation of the mean for repeat experiments. **E** summary of amphiphysin function during cytokinesis and cell separation. Anaphase B: Rvs167/161 (indicated in blue) and Iqg1 localise to the division site promoting CAR formation (purple circles). The SH3 domain of Rvs167 is required for this process. Spindle disassembly marks the start of telophase. Shortly thereafter CAR contraction is initiated driving centripetal invagination of the plasma membrane behind which the primary septum is formed (green bar). Concomitantly, Rvs167/161 patches (blue) concentrate at the division site triggering polarity establishment (red arrows). This is dependent upon the amphiphysin BAR domains that manipulate membrane curvature and microdomain lipid composition. Polarity establishment then drives assembly of septum formation specific protein complexes. Rvs167 is required for the polarised accumulation of active Rho1-GTP and its effector Chs3 at the bud neck and also mediates correct localisation of the Rho1 GEF, Rom2, during secondary septum formation (yellow bars). These and likely additional factors promote septum formation. Rvs167/161 remains associated at the division site following cell separation.

Secondary septum deposition and cell wall synthesis requires polarised assembly and activation of the relevant enzyme activities requiring catalytic components, Chs3 and Fks1 respectively. Both are effectors of activated, GTP bound, Rho1, which is localized to the plasma membrane at the bud neck through interaction between the poly-basic Rho1 C-terminus and PIP2 [25]. Loss of amphiphysin function results in altered localization of the Rho1-GEF, Rom2 (Fig. 8). Potentially this would result in a failure to generate GTP bound Rho1 within the appropriate temporal-spatial window and a consequent failure to accumulate Rho1-GTP at the bud neck at the point of cell separation. The data presented in Fig. 9 demonstrate that this localization is completely abolished in the *rvs167* null mutant. Both of these consequences, failure to properly localize Rom2 and Rho1, might stem from reduced levels of PIP2 at the bud neck that might be dependent upon Rvs167 plasma membrane binding analogous to one suggested role of BAR domain proteins in endocytic mechanisms [30]. Further support for this line of reasoning derives from recent evidence that has clearly demonstrated that budding yeast BAR domain proteins, including Rvs167, are able to organise stable, discrete PIP_2_ domains within the plasma membrane that allow for the assembly of plasma membrane associated protein complexes [64]. This interpretation does not exclude the possibility of direct interaction between the amphiphysin complex and either Rom2 or Rho1 although neither protein has previously been reported to interact with Rvs167 despite extensive interaction screens recorded in the *Saccharomyces* Genome database. Importantly though, to the best of our knowledge, these data represent the first demonstration of an amphiphysin complex acting upstream in the activation of a Rho family GTPase.

In conclusion, the amphiphysin complex has two major functions during cytokinesis and cell separation in budding yeast as summarised in Fig. 9E, firstly, an auxilliary role in CAR assembly during early mitosis that requires the SH3 domain and is redundant with Hof1 [50]. Secondly, when CAR contraction is compromised, the amphiphysin polarity establishment function is required for the correct localization of at least three proteins, Rom2, Chs3 and GTP-bound Rho1 that function at the bud neck during cytokinesis and cell separation. The combination of failed CAR contraction and perturbation of polarity establishment leads to synthetic lethality. That Rvs167 is involved in both processes may serve to increase co-ordination between them, with subsequent gains in efficiency. The clear redundancy exhibited, both within and between CAR assembly and septation/cell separation, imply that CAR contraction itself might play a role in polarity establishment. Further, it is likely that this redundancy reflects evolution of robustness in the process of cytokinesis and cell separation.

## Materials and Methods

### Yeast strains, methods, and media

The yeast strains used in this study were isogenic with W303a and are listed in Table 1. Growth was in YEPD at the stated temperature [55]. Strains for fluorophore visualization were grown in complete or selective synthetic medium [55]. Solid media was made to the same composition, but with the addition of 2% agar. Salt sensitivity was tested by the addition of 6% sodium chloride. For positive selection of strains harbouring the drug resistance markers *kanMX6*, *hphMX6* or *natMX6* YEPD agar was supplemented with 220 µg/ml G418 disulphate (Melford), 300 µg/ml hygromycin B (InvivoGen), or 100 µg/ml nourseothricin (Werner Bioagents) respectively. Yeast transformations were carried out by a standard lithium acetate method [56]. *GAL-CDC20*, *cdc20Δ* cells were grown to exponential phase in SC Gal/Raf (as above, but substituting 2% galactose and 4% raffinose for glucose), before being transferred to SC containing 2% glucose to induce metaphase arrest. Release from the arrest was achieved by washing cells in 10 culture volumes of selective SC Gal/Raf and then resuspending cells in 1 volume of SC Gal/Raf. Induction of *MET3* promoters was performed by growing cells in selective synthetic media lacking methionine. Repression of *MET3* promoters was achieved by addition of sterile methionine to cultures (at a final amount of 50 mg/litre).

**Table 1:** 

Strain number	Genotype	Source
SSC1	*MAT* **a** *ade2-1 his3-11,15 leu2-3,112 ura3-1 trp1-1 can1-100*	Laboratory collection
SSC2	*MAT*α *ade2-1 his3-11,15 leu2-3,112 ura3-1 trp1-1 can1-100*	Laboratory collection
SSC3	*MAT* **a**/α *ade2-1/ade2-1 his3-11,15/his3-11,15 leu2-3,112/leu2-3,112 ura3-1/ura3-1 trp1-1/trp1-1 can1-100/can1-100*	Laboratory collection
SSC166	*MAT* **a** *ade2-1 his3-11,15 leu2-3,112 ura3-1 trp1-1 can1-100 iqg1-1*	Laboratory collection
SSC619	*MAT* **a** *ade2-1 his3-11,15 leu2-3,112 ura3-1 trp1-1 can1-100 rvs167Δ::TRP1*	K. Ayscough
SSC1001	*MAT* **a**/α *ade2-1/ade2-1 his3-11,15/his3-11,15 leu2-3,112/leu2-3,112 ura3-1/ura3-1 trp1-1/trp1-1 can1-100/can1-100 iqg1-1/IQG1 rvs167Δ::TRP1/RVS167*	This study
SSC1614	*MAT* **a** *ade2-1 his3-11,15 leu2-3,112 ura3-1 trp1-1 can1-100 RVS167-GFP::natMX6*	This study
SSC1636	*MAT* **a**/α *ade2-1/ade2-1 his3-11,15/his3-11,15 leu2-3,112/leu2-3,112 ura3-1/ura3-1 trp1-1/trp1-1 can1-100/can1-100 iqg1-1/IQG1 rvs161Δ::kanMX4/RVS161*	This study
SSC1659	*MAT* **a** *ade2-1 his3-11,15 leu2-3,112 ura3-1 trp1-1 can1-100 RVS167-GFP::natMX6 iqg1-1*	This study
SSC1754	*MAT* **a** *ade2-1 his3-11,15 ura3-1 trp1-1 can1-100 leu2-3,112::YIplac128-pMET3-RVS167-13MYC::LEU2 rvs167Δ::TRP1*	This study
SSC1756	*MAT* **a** *ade2-1 his3-11,15 ura3-1 trp1-1 can1-100 leu2-3,112::YIplac128-pMET3-RVS167-13MYC::LEU2 iqg1-1*	This study
SSC1758	*MAT* **a** *ade2-1 his3-11,15 ura3-1 trp1-1 can1-100 leu2-3,112::YIplac128-pMET3-RVS167-13MYC::LEU2 rvs167Δ::TRP1 iqg1-1*	This study
SSC1767	*MAT* **a** *ade2-1 his3-11,15 ura3-1 trp1-1 can1-100 leu2-3,112::YIplac128-pMET3-RVS167-13MYC::LEU2*	This study
SSC1792	*MAT* **a** *ade2-1 his3-11,15 leu2-3,112 ura3-1 trp1-1 can1-100 IQG1-VC155::kanMX6 RVS167-VN173::His3MX6*	This study
SSC1794	*MAT* **a** *ade2-1 his3-11,15 leu2-3,112 ura3-1 trp1-1 can1-100 RVS167-VC155::kanMX6 BSP1-VN173::His3MX6*	This study
SSC1853	*MAT* **a** *ade2-1 his3-11,15 ura3-1 trp1-1 can1-100 leu2-3,112::YIplac128-pMET3-RVS167-13MYC::LEU2 MYO1-GFP::natMX6 rvs167Δ::TRP1*	This study
SSC1854	*MAT* **a** *ade2-1 his3-11,15 ura3-1 trp1-1 can1-100 leu2-3,112::YIplac128-pMET3-RVS167-13MYC::LEU2 MYO1-GFP::natMX6 iqg1-1*	This study
SSC1855	*MAT* **a** *ade2-1 his3-11,15 ura3-1 trp1-1 can1-100 leu2-3,112::YIplac128-pMET3-RVS167-13MYC::LEU2 MYO1-GFP::natMX6 rvs167Δ::TRP1 iqg1-1*	This study
SSC1859	*MAT* **a** *ade2-1 his3-11,15 ura3-1 trp1-1 can1-100 leu2-3,112::YIplac128-pMET3-RVS167-13MYC::LEU2 MYO1-GFP::natMX6*	This study
SSC1987	*MAT* **a**/α *ade2-1/ade2-1 his3-11,15/his3-11,15 leu2-3,112/leu2-3,112 ura3-1/ura3-1 trp1-1/trp1-1 can1-100/can1-100 RVS167-VC155::kanMX6/RVS167-VN173::His3MX6*	This study
SSC2070	*MAT* **a** *ade2-1 his3-11,15 leu2-3,112 ura3-1 trp1-1 can1-100 RVS167-VC155::kanMX6 CYK3-VN173::His3MX6*	This study
SSC2072	*MAT* **a** *ade2-1 his3-11,15 leu2-3,112 ura3-1 trp1-1 can1-100 RVS167-VC155::kanMX6 HOF1-VN173::His3MX6*	This study
SSC2256	*MAT* **a** *ade2-1 his3-11,15 leu2-3,112 ura3-1 trp1-1 can1-100 RVS167-VC155::kanMX6 CYK3-VN173::His3MX6 bsp1Δ::His3MX6*	This study
SSC2258	*MAT* **a** *ade2-1 his3-11,15 leu2-3,112 ura3-1 trp1-1 can1-100 RVS167-VC155::kanMX6 CYK3-VN173::His3MX6 hof1Δ::kanMX6*	This study
SSC2293	*MAT* **a** *ade2-1 his3-11,15 leu2-3,112 ura3-1 trp1-1 can1-100 RVS161-VC155::kanMX6 IQG1-VN173::His3MX6*	This study
SSC2296	*MAT* **a** *ade2-1 his3-11,15 leu2-3,112 ura3-1 trp1-1 can1-100 RVS161-VC155::kanMX6 RVS167-VN173::His3MX6*	This study
SSC2299	*MAT* **a** *ade2-1 his3-11,15 leu2-3,112 ura3-1 trp1-1 can1-100 RVS161-VC155::kanMX6 HOF1-VN173::His3MX6*	This study
SSC2302	*MAT* **a** *ade2-1 his3-11,15 leu2-3,112 ura3-1 trp1-1 can1-100 RVS161-VC155::kanMX6 CYK3-VN173::His3MX6*	This study
SSC2305	*MAT* **a** *ade2-1 his3-11,15 leu2-3,112 ura3-1 trp1-1 can1-100 RVS161-VC155::kanMX6 BSP1-VN173::His3MX6*	This study
SSC2315	*MAT* **a** *ade2-1 his3-11,15 leu2-3,112 ura3-1 trp1-1 can1-100 RVS167-VC155::kanMX6 CYK3-VN173::His3MX6 rvs161Δ::natMX6*	This study
SSC2698	*MAT* **a** *ade2-1 his3-11,15 leu2-3,112 ura3-1 trp1-1 can1-100 TPM2-GFP::natMX6 [YCplac33-pMET3-eCFP-TUB3::URA3]*	This study
SSC2699	*MAT* **a** *ade2-1 his3-11,15 leu2-3,112 ura3-1 trp1-1 can1-100 TPM2-GFP::natMX6 [YCplac33-pMET3-eCFP-TUB3::URA3] rvs167Δ::TRP1*	This study
SSC2700	*MAT* **a** *ade2-1 his3-11,15 leu2-3,112 ura3-1 trp1-1 can1-100 TPM2-GFP::natMX6 [YCplac33-pMET3-eCFP-TUB3::URA3] iqg1-1*	This study
SSC2724	*MAT* **a** *ade2-1 his3-11,15 ura3-1 trp1-1 can1-100 leu2-3,112::YIplac128-pMET3-RVS167-13MYC::LEU2 INN1-GFP::natMX6*	This study
SSC2725	*MAT* **a** *ade2-1 his3-11,15 ura3-1 trp1-1 can1-100 leu2-3,112::YIplac128-pMET3-RVS167-13MYC::LEU2 INN1-GFP::natMX6 rvs167Δ::TRP1*	This study
SSC2727	*MAT* **a** *ade2-1 his3-11,15 ura3-1 trp1-1 can1-100 leu2-3,112::YIplac128-pMET3-RVS167-13MYC::LEU2 INN1-GFP::natMX6 iqg1-1*	This study
SSC2729	*MAT* **a** *ade2-1 his3-11,15 ura3-1 trp1-1 can1-100 leu2-3,112::YIplac128-pMET3-RVS167-13MYC::LEU2 INN1-GFP::natMX6 rvs167Δ::TRP1 iqg1-1*	This study
SSC2735	*MAT* **a** *ade2-1 his3-11,15 leu2-3,112 ura3-1 trp1-1 can1-100 GAL-CDC20::TRP1 cdc20Δ::LEU2 RVS167-GFP::natMX6 MYO1-tdTomato::kanMX6 SPC29-tdTomato::hphMX6*	This study
SSC2829	*MAT* **a** *ade2-1 his3-11,15 leu2-3,112 ura3-1 trp1-1 can1-100 RVS167-VC155::kanMX6 INN1-VN173::His3MX6*	This study
SSC2832	*MAT* **a** *ade2-1 his3-11,15 leu2-3,112 ura3-1 trp1-1 can1-100 RVS161-VC155::kanMX6 INN1-VN173::His3MX6*	This study
SSC2876	*MAT* **a** *ade2-1 his3-11,15 leu2-3,112 ura3-1 trp1-1 can1-100 RVS167^ΔSH3^-GFP::natMX6*	This study
SSC2880	*MAT* **a** *ade2-1 his3-11,15 leu2-3,112 ura3-1 trp1-1 can1-100 RVS167^ΔSH3^-GFP::natMX6 iqg1-1*	This study
SSC2942	*MAT* **a** *ade2-1 his3-11,15 trp1-1 can1-100 leu2-3,112::YIplac128-pMET3-RVS167-13MYC::LEU2 ura3-1::YIplac211-pGAL1-eCFP-TUB3::URA3 TPM2-GFP::natMX6*	This study
SSC2944	*MAT* **a** *ade2-1 his3-11,15 trp1-1 can1-100 leu2-3,112::YIplac128-pMET3-RVS167-13MYC::LEU2 ura3-1::YIplac211-pGAL1-eCFP-TUB3::URA3 TPM2-GFP::natMX6 rvs167Δ::TRP1*	This study
SSC2946	*MAT* **a** *ade2-1 his3-11,15 trp1-1 can1-100 leu2-3,112::YIplac128-pMET3-RVS167-13MYC::LEU2 ura3-1::YIplac211-pGAL1-eCFP-TUB3::URA3 TPM2-GFP::natMX6 iqg1-1*	This study
SSC2948	*MAT* **a** *ade2-1 his3-11,15 trp1-1 can1-100 leu2-3,112::YIplac128-pMET3-RVS167-13MYC::LEU2 ura3-1::YIplac211-pGAL1-eCFP-TUB3::URA3 TPM2-GFP::natMX6 rvs167Δ::TRP1 iqg1-1*	This study
SSC2759	*MAT a ade2-1 his3-11,15 ura3-1 trp1-1 can1-100 leu2-3,112::YIplac128-pMET3-RVS167-13MYC::LEU2 [pYEURA3-GAL1,10-MLC1::URA3]*	This study
SSC2760	*MAT a ade2-1 his3-11,15 ura3-1 trp1-1 can1-100 leu2-3,112::YIplac128-pMET3-RVS167-13MYC::LEU2 [pYEURA3-GAL1,10-MLC1::URA3] rvs167Δ::TRP1*	This study
SSC2761	*MAT a ade2-1 his3-11,15 ura3-1 trp1-1 can1-100 leu2-3,112::YIplac128-pMET3-RVS167-13MYC::LEU2 [pYEURA3-GAL1,10-MLC1::URA3] iqg1-1*	This study
SSC2762	*MAT a ade2-1 his3-11,15 ura3-1 trp1-1 can1-100 leu2-3,112::YIplac128-pMET3-RVS167-13MYC::LEU2 [pYEURA3-GAL1,10-MLC1::URA3] rvs167Δ::TRP1 iqg1-1*	This study
SSC2090	*MAT a ade2-1 his3-11,15 ura3-1 trp1-1 can1-100 leu2-3,112::YIplac128-pMET3-RVS167-13MYC::LEU2 [pYES2.0-GAL1-BSP1::URA3]*	This study
SSC2091	*MAT a ade2-1 his3-11,15 ura3-1 trp1-1 can1-100 leu2-3,112::YIplac128-pMET3-RVS167-13MYC::LEU2 [pYES2.0-GAL1-BSP1::URA3] rvs167Δ::TRP1*	This study
SSC2092	*MAT a ade2-1 his3-11,15 ura3-1 trp1-1 can1-100 leu2-3,112::YIplac128-pMET3-RVS167-13MYC::LEU2 [pYES2.0-GAL1-BSP1::URA3] iqg1-1*	This study
SSC2093	*MAT a ade2-1 his3-11,15 ura3-1 trp1-1 can1-100 leu2-3,112::YIplac128-pMET3-RVS167-13MYC::LEU2 [pYES2.0-GAL1-BSP1::URA3] rvs167Δ::TRP1 iqg1-1*	This study
SSC2743	*MAT*α *ade2-1 his3-11,15 leu2-3,112 ura3-1 trp1-1 can1-100 [YEplac181-pMET3-BSP1::LEU2]*	This study
SSC2744	*MAT*α *ade2-1 his3-11,15 leu2-3,112 ura3-1 trp1-1 can1-100 [YEplac181-pMET3-BSP1::LEU2] abp1Δ::URA3*	This study
SSC2745	*MAT*α *ade2-1 his3-11,15 leu2-3,112 ura3-1 trp1-1 can1-100 [YEplac181-pMET3-BSP1::LEU2] iqg1-1*	This study
SSC2746	*MAT*α *ade2-1 his3-11,15 leu2-3,112 ura3-1 trp1-1 can1-100 [YEplac181-pMET3-BSP1::LEU2] abp1Δ::URA3 iqg1-1*	This study
SSC2183	*MATa ade2-1 his3-11,15 leu2-3,112 trp1-1 can1-100 ura3-1::YIplac211-pMET3-eCFP-TUB3::URA3 BSP1-GFP::natmx6*	This study
SSC2186	*MATa ade2-1 his3-11,15 leu2-3,112 trp1-1 can1-100 ura3-1::YIplac211-pMET3-eCFP-TUB3::URA3 BSP1-GFP::natmx6 rvs167Δ::TRP1 rvs161Δ::hphMX6*	This study
SSC1016	*MATa ade2-1 his3-11,15 leu2-3,112 ura3-1 trp1-1 can1-100 CHS3-GFP(S65T)::KanMX6*	This study
SSC2055	*MATa ade2-1 his3-11,15 leu2-3,112 ura3-1 trp1-1 can1-100 CHS3-GFP(S65T)::KanMX6 rvs167Δ::TRP1 rvs161Δ::natMX6*	This study
SSC2531	*MATa ade2-1 his3-11,15 leu2-3,112 ura3-1 trp1-1 can1-100 [YCplac111-pMET3-eCFP-TUB3::LEU2] [GFP-RHO1::URA3]*	This study
SSC2532	*MATa ade2-1 his3-11,15 leu2-3,112 ura3-1 trp1-1 can1-100 [YCplac111-pMET3-eCFP-TUB3::LEU2] [GFP-RHO1::URA3] iqg1-1*	This study
SSC2533	*MATa ade2-1 his3-11,15 leu2-3,112 ura3-1 trp1-1 can1-100 [YCplac111-pMET3-eCFP-TUB3::LEU2] [GFP-RHO1::URA3] rvs167Δ::TRP1*	This study
SSC2541	*MATa ade2-1 his3-11,15 leu2-3,112 ura3-1 trp1-1 can1-100 [YCplac111-pMET3-eCFP-TUB3::LEU2] [GFP-RHO1^Q68H^::URA3]*	This study
SSC2542	*MATa ade2-1 his3-11,15 leu2-3,112 ura3-1 trp1-1 can1-100 [YCplac111-pMET3-eCFP-TUB3::LEU2] [GFP-RHO1^Q68H^::URA3] iqg1-1*	This study
SSC2543	*MATa ade2-1 his3-11,15 leu2-3,112 ura3-1 trp1-1 can1-100 [YCplac111-pMET3-eCFP-TUB3::LEU2] [GFP-RHO1^Q68H^::URA3] rvs167Δ::TRP1*	This study
SSC1890	*MATa ade2-1 his3-11,15 leu2-3,112 ura3-1 trp1-1 can1-100 [YCplac33-pMET3-eCFP-TUB3::URA3] Cyk3-GFP::KanMX*	This study
SSC2146	*MATa ade2-1 his3-11,15 leu2-3,112 ura3-1 trp1-1 can1-100 [YCplac33-pMET3-eCFP-TUB3::URA3] Cyk3-GFP::KanMX rvs167Δ::TRP1*	This study
SSC2868	*MAT*α *ade2-1 his3-11,15 ura3-1 trp1-1 can1-100 leu2-3,112::pMET3::YIplac128-pMET3-eCFP-TUB3::LEU2 HOF1-GFP::kanMX6*	This study
SSC2870	*MAT*α *ade2-1 his3-11,15 ura3-1 trp1-1 can1-100 leu2-3,112::pMET3::YIplac128-pMET3-eCFP-TUB3::LEU2 HOF1-GFP::kanMX6 rvs167Δ::TRP1*	This study
SSC2487	*MATa ade2-1 his3-11,15 leu2-3,112 ura3-1 trp1-1 can1-100 Rom2-GFP::natMX6*	This study
SSC2662	*MATa ade2-1 his3-11,15 leu2-3,112 ura3-1 trp1-1 can1-100 Rom2-GFP::natMX6 rvs167Δ::TRP1*	This study
SSC2664	*MATa ade2-1 his3-11,15 leu2-3,112 ura3-1 trp1-1 can1-100 Rom2-GFP::natMX6 iqg1-1*	This study
SSC2484	*MATa ade2-1 his3-11,15 leu2-3,112 ura3-1 trp1-1 can1-100 Tus1-GFP::natMX6*	This study
SSC2649	*MATa ade2-1 his3-11,15 leu2-3,112 ura3-1 trp1-1 can1-100 Tus1-GFP::natMX6 rvs167Δ::TRP1*	This study
SSC2651	*MATa ade2-1 his3-11,15 leu2-3,112 ura3-1 trp1-1 can1-100 Tus1-GFP::natMX6 iqg1-1*	This study

### Plasmid construction

Standard DNA manipulations were employed for plasmid construction () with all DNA modifying enzymes used according to manufacturers specifications. The *Escherichia coli* strains, TOP10 (Invitrogen) or DH5α (Bioline) were used as a host for plasmid amplifications. Bacterial strains were grown in 2×TY media and frozen competent cells transformed according to manufacturers instructions. Plasmids used in this study are listed in Table 2.

**Table 2:** 

Plasmid number	Plasmid and (construction)	Source
DNA425	*YIplac128-pMET3::LEU2*	Laboratory collection
DNA465	*pFA6a-13MYC::kanMX6*	[57]
DNA614	*pFA6a-tdTomato::kanMX6*	Iain Hagan
DNA615	*pFA6a-tdTomato::hphMX6*	Iain Hagan
DNA637	*pFA6a-GFP(S65T)::natMX6*	[59]
DNA669	*YCplac33-pMET3-eCFP-TUB3::URA3*	This study
DNA679	*YIplac128-pMET3-RVS167-13MYC::LEU2*	This study
DNA689	*pFA6a-VC155::kanMX6*	[60]
DNA690	*pFA6a-VN173::His3MX6*	[60]
DNA734	*YIplac211-pGAL1-eCFP-TUB3::URA3*	This study
DNA317	*pYEURA3-GAL1,10-MLC1::URA3*	Laboratory collection
DNA713	*pYES2.0-GAL1-BSP1::URA3*	This study
DNA484	*YEplac181-pMET3-BSP1::LEU2*	This study
DNA667	*YIplac211-pMET3-eCFP-TUB3::URA3*	This study
DNA619	*pFA6a::hphMX6*	[59]
DNA463	*pFA6a-GFP(S65T)::kanMX6*	[57]
DNA618	*pFA6a::natMX6*	[59]
DNA754	*GFP-RHO1::URA3*	SP301 [29]
DNA756	*GFP-RHO1^Q68H^::URA3*	SP307 [29]
DNA666	*YIplac128-pMET3-eCFP-TUB3::LEU2*	This study

### Epitope tagging and strain construction

Epitope tagging was carried out by standard PCR amplification and subsequent homologous recombination into the yeast genome at the endogenous locus [57]. Further tagging and deletion of genes was similarly achieved using the plasmids pFA6a-GFP-kanMX6, pFA6a-HIS3-kanMX6, pFA6a-CFP-kanMX6, pFA6a-YFP-kanMX6, pFA6a-hphMX6, pFA6a-VC155::kanMX6 and pFA6a-VN173::*HIS3*MX6 as templates with appropriate primers [57], [58], [59], [60]. Predicted sites of integration at the endogenous locus were confirmed by PCR testing of linkage of the drug resistant marker to flanking genomic DNA sequences. Yeast genomic DNA was isolated by a standard protocol [61]. All relevant DNA primer sequences are available on request.

In order to create the methionine regulated *RVS167-13myc strains the RVS167* was first epitope tagged by standard methodologies. The *RVS167-13MYC* allele was then amplified by PCR and inserted into *pCR-BluntII-TOPO* (Invitrogen) according to manufacturer's instructions. *RVS167-13MYC* was excised as a Xho1/BglII fragment and ligated into SalI/BamHI cut *YIplac128-pMET3 (this work) to generate YIplac128-pMET3-RVS167-13MYC*. The plasmid was linearized by restriction digest with XcmI and integrated at the LEU2 locus to create the strain SSC1767. Subsequently the endogenous RVS167 was deleted by gene replacement with *TRP1* to create SSC1754. An identical approach was used to create the integrated, methionine regulated RVS161-13myc allele in strains.

### Preparation and staining of fixed cells for epifluorescence microscopy

Actin staining: 1×10^8^ formaldehyde fixed cells harvested by centrifugation at 4,000 rpm, resuspended in 25 µl of PBS solution containing 3 µm TRITC-conjugated phalloidin (Sigma) and incubated in the dark at room temperature for 90 minutes [62]. Nuclear staining: cells were harvested as above and resuspended in an appropriate volume of Vectashield with DAPI (Vector Laboratories). Cells were then either stored at 4°C or prepared immediately for microscopy.

### Microscopy: image acquisition and analysis

Cells were prepared for live cell microscopy and images acquired and analysed as previously described [63].

## Supporting Information

Figure S1 **Rvs167-13Myc is functional.** Indicated strains were grown to mid-log phasein media lacking methionine before plating onto indicated media and grown at temperature shown before imaging. A. Ectopic Rvs167-Myc expression rescues rvs167Δ iqg1-1 synthetic lethality (48 h growth). B. Ectopic Rvs167-Myc expression rescues salt sensitivity associated with rvs167Δ (128 h growth). C. Rvs167-Myc expression rescues actin polarity defects associated with rvs167**Δ**. Cells with ≥80% of actin patches localised in the bud or immediately adjacent at the bud neck were scored as polarised (between 94–145 cells scored at each time point). Shown are representative images from −MET (T = 0) of WT cells, with polarised actin patches, and *rvs167* **Δ** cells with polarised (−MET) and depolarised (+MET) patches respectively. eV = ‘empty’ control vector. Scale bars = 2.5 um. **D**. Ectopic Rvs161-Myc expression rescues *rvs161* **Δ** *iqg1-1* synthetic lethality. E. Ectopic Rvs161-Myc expression only partially restores *rvs161*Δ salt sensitivity after 128 h growth.
(TIF)

Figure S2 **Cyk3 and Hof1 behaviour is unaltered in the absence of amphiphysin function.** (A) CYK3-GFP localisation is unaltered in rvs167**Δ** cells as shown by representative average intensity fluorescent images (scale bars = 2 **µ**m). Maximum projected reconstructions of pixel data at the bud neck demonstrate Cyk3-GFP localises at ring and septal structures (scale bars for reconstructions = 1 **µ**m). (B) Quantification of Cyk3-GFP localization in large budded anaphase WT (n = 111), *rvs167* **Δ** (n = 66), *rvs161* **Δ** (n = 99), *rvs167* **Δ** *rvs161* **Δ** (n = 106) cells (left graph) and telophase WT (n = 130), *rvs167* **Δ** (n = 173), *rvs161* **Δ** (n = 148), *rvs167* **Δ** *rvs161* **Δ** (n = 135) cells (right graph). (C) Hof1-GFP localisation is unaltered in rvs167**Δ** cells. Fluorescent panels represent average intensity projections (scale bars = 2.5 **µ**m), except bud neck reconstructions (maximum projections, scale bars 1 **µ**m) that show Hof1-GFP localisation to rings in anaphase and telophase and to septa in late telophase. Cyk3-GFP (A) and Hof1-GFP (C) both form split bands either side of the division site in late telophase (arrowheads).
(TIF)

Figure S3 **Tus1-GFP dynamics are normal in iqg1-1 and rvs167Δ mutants.** WT (A), *iqg1-1* (B) and *rvs167* **Δ** (C) cells expressing Tus1-GFP were imaged at 1 minute intervals (3 second exposures, 18 z-sections, 0.2 **µ**m z-spacing) for 35 minutes. Maximum intensity projections of deconvolved z-stacks are shown for indicated time points.
(TIF)

## References

[1] Barr FA, Gruneberg U (2007) Cytokinesis: placing and making the final cut. Cell 131: 847–860. 18045532 10.1016/j.cell.2007.11.011

[2] Pollard TD (2010) Mechanics of cytokinesis in eukaryotes. Curr Opin in Cell Biology 22: 50–56. 10.1016/j.ceb.2009.11.010 PMC2871152 20031383

[3] Schmidt M, Bowers B, Varma A, Roh D-H, Cabib E (2002) In budding yeast, contraction of the actomyosin ring and formation of the primary septum at cytokinesis depend on each other. J Cell Sci 115: 293–302. 11839781 10.1242/jcs.115.2.293

[4] VerPlank L, Li R (2005) Cell cycle-regulated trafficking of Chs2 controls acto-myosin ring stability during cytokinesis. Mol Biol Cell 16: 2529–2543. 15772160 10.1091/mbc.E04-12-1090 PMC1087255

[5] Bi E, Park H-O (2012) Cell polarization and cytokinesis in budding yeast. Genetics 191: 347–387. 22701052 10.1534/genetics.111.132886 PMC3374305

[6] Shaw JA, Mol PC, Bowers B, Silverman SJ, Valdivieso MH, et al (1991) The function of chitin synthases 2 and 3 in the *Saccharomyces* cerevisiae cell cycle. J Cell Biol 114: 111–123. 2050738 10.1083/jcb.114.1.111 PMC2289062

[7] Lesage G, Bussey H (2006) Cell wall assembly in *Saccharomyces cerevisiae* . Microbiol Mol Biol Rev 70: 317–343. 16760306 10.1128/MMBR.00038-05 PMC1489534

[8] Kuranda MJ, Robbins PW (1991) Chitinase is required for cell separation during growth of *Saccharomyces cerevisiae* . J Biol Chem 266: 19758–19767. 1918080

[9] Epp JA, Chant J (1997) An IQGAP-related protein controls actin-ring formation and cytokinesis in yeast. Curr Biol 7: 921–929. 9382845 10.1016/s0960-9822(06)00411-8

[10] Lippincott J, Li R (1998a) Sequential assembly of myosin II, an IQGAP-like protein, and filamentous actin to a ring structure involved in budding yeast cytokinesis. J Cell Biol 140: 355–366. 9442111 10.1083/jcb.140.2.355 PMC2132585

[11] Kamei T, Tanaka K, Hihara T, Umikawa M, Imamura H, et al (1998) Interaction of Bnr1p with a novel Src homology 3 domain-containing Hof1p. J Biol Chem 273: 28341–28345. 9774458 10.1074/jbc.273.43.28341

[12] Korinek WS, Bi E, Epp JA, Wang L, Ho J, et al (2000) Cyk3, a novel SH3-domain protein, affects cytokinesis in yeast. Curr Biol 10: 947–950. 10959846 10.1016/s0960-9822(00)00626-6

[13] Sanchez-Diaz A, Marchesi V, Murray S, Jones R, Pereira G, et al (2008) Inn1 couples contraction of the actomyosin ring to membrane ingression during cytokinesis in budding yeast. Nat Cell Biol 10: 395–406. 18344988 10.1038/ncb1701

[14] Lippincott J, Li R (1998b) Dual function of Cyk2, a cdc15/PSTPIP family protein in regulating actomyosin ring dynamics and septin distribution. J Cell Biol 143: 1947–1960. 9864366 10.1083/jcb.143.7.1947 PMC2175218

[15] Shannon KB, Li R (1999) The multiple roles of Cyk1p in the assembly and function of the actomyosin ring in budding yeast. Mol Biol Cell 10: 283–296. 9950677 10.1091/mbc.10.2.283 PMC25169

[16] Boyne JR, Yosuf HM, Bieganowski P, Brenner C, Price C (2000) Yeast myosin light chain, Mlc1p, interacts with both IQGAP and class II myosin to effect cytokinesis. J Cell Sci 113: 4533–4543. 11082046 10.1242/jcs.113.24.4533

[17] Corbett M, Xiong Y, Boyne JR, Wright DJ, Munro E, et al (2006) IQGAP and mitotic exit network (MEN) proteins are required for cytokinesis and re-polarization of the actin cytoskeleton in the budding yeast, *Saccharomyces cerevisiae* . Eur J Cell Biol 11: 1201–1215. 10.1016/j.ejcb.2006.08.001 17005296

[18] Jendretzki A, Ciklic I, Rodicio R, Schmitz HP, Heinisch JJ (2009) Cyk3 acts in actomyosin ring independent cytokinesis by recruiting Inn1 to the yeast bud neck. Mol Genet Genomics 282: 437–51. 19707790 10.1007/s00438-009-0476-0

[19] Nishihama R, Schreiter JH, Onishi M, Vallen EA, Hanna J, et al (2009) Role of Inn1 and its interactions with Hof1 and Cyk3 in promoting cleavage furrow and septum formation in *S. cerevisiae* . J Cell Biol 185: 995–1012. 19528296 10.1083/jcb.200903125 PMC2711614

[20] Meitinger F, Petrova B, Mancini Lombardi I, Trinca Bertazzi D, Hub B, et al (2010) Targeted localization of Inn1, Cyk3 and Chs2 by the mitotic-exit network regulates cytokinesis in budding yeast. J Cell Sci 123: 1851–1861. 20442249 10.1242/jcs.063891

[21] Fang X, Luo J, Nishihama R, Wloka C, Dravis C, et al (2010) Biphasic targeting and cleavage furrow ingression directed by the tail of a myosin II. J Cell Biol 191: 1333–1350. 21173112 10.1083/jcb.201005134 PMC3010076

[22] Palani S, Meitinger F, Boehm ME, Lehmann WD, Pereira G (2012) Cdc14-dependent dephosphorylation of Inn1 contributes to Inn1-Cyk3 complex formation. J Cell Sci 125: 3091–3096. 22454527 10.1242/jcs.106021

[23] Tolliday N, VerPlank L, Li R (2002) Rho1 directs forming mediated actin ring assembly during budding yeast cytokinesis. Curr Biol 12: 1864–1870. 12419188 10.1016/s0960-9822(02)01238-1

[24] Yoshida S, Kono K, Lowery DM, Bartolini S, Yaffe MB, et al (2006) Polo-like kinase Cdc5 controls the local activation of Rho1 to promote cytokinesis. Science 313: 108–111. 16763112 10.1126/science.1126747

[25] Yoshida S, Bartolini S, Pellman D (2009) Mechanisms for concentrating Rho1during cytokinesis. Genes and Dev 23: 810–823. 19339687 10.1101/gad.1785209 PMC2666341

[26] Onishi M, Ko N, Nishihama R, Pringle JR (2013) Distinct roles of Rho1, Cdc42 and Cyk3 in septum formation and abscission during yeast cytokinesis. J Cell Biol 202: 311–329. 23878277 10.1083/jcb.201302001 PMC3718969

[27] Munn AL, Stevenson BJ, Geli M, Riezman H (1995) end5, end6, and end7: mutations that cause actin delocalization and block the internalization step of endocytosis in *Saccharomyces cerevisiae* . Mol Biol Cell 6: 1721–42. 8590801 10.1091/mbc.6.12.1721 PMC301328

[28] Sanchez-Diaz A, Nkosi PJ, Murray S, Labib K (2012) The mitotic exit network and Cdc14 phosphatase initiate cytokinesis by counteracting CDK phosphorylations and blocking polarized growth. EMBO J 31: 3620–3634. 22872148 10.1038/emboj.2012.224 PMC3433788

[29] Wright DJ, Munro E, Corbett M, Bentley AJ, Fullwood NJ, et al (2008) The *Saccharomyces cerevisiae* actin cytoskeletal component Bsp1p has an auxiliary role in actomyosin ring function and in the maintenance of bud neck structure. Genetics 178: 1903–1914. 18430924 10.1534/genetics.107.082685 PMC2323785

[30] Liu J, Sun Y, Drubin DG, Oster GF (2009) The mechanochemistry of endocytosis. PLoS Biol 7: e1000204. 19787029 10.1371/journal.pbio.1000204 PMC2742711

[31] Galletta BJ, Mooren OL, Cooper JA (2010) Actin dynamics and endocytosis in yeast and mammals. Curr Op in Biotech 21: 604–610. 10.1016/j.copbio.2010.06.006 PMC2952672 20637595

[32] Peter BJ, Kent HM, Mills IG, Vallis Y, Butler PJ, et al (2004) BAR domains as sensors of membrane curvature: the amphiphysin BAR structure. Science 303: 495–499. 14645856 10.1126/science.1092586

[33] Youn J-Y, Friesen H, Kishimoto T, Henne WM, Kurat CF, et al (2010) Dissecting BAR domain function in the yeast amphiphysins Rvs161 and Rvs167 during endocytosis. Mol Biol Cell 21: 3054–3069. 20610658 10.1091/mbc.E10-03-0181 PMC2929998

[34] Bauer F, Urdaci M, Aigle M, Crouzet M (1993) Alteration of a yeast SH3 protein leads to conditional viability with defects in cytoskeletal and budding patterns. Mol Cell Biol 13: 5070–5084. 8336735 10.1128/mcb.13.8.5070-5084.1993 PMC360159

[35] Sivadon P, Bauer F, Aigle M, Crouzet M (1995) Actin cytoskeleton and budding pattern are altered in the yeast *rvs161* mutant: the Rvs161 protein shares common domains with the brain protein amphiphysin. Mol Gen Genet 246: 485–495. 7891662 10.1007/BF00290452

[36] Sivadon P, Crouzet M, Aigle M (1997) Functional assessment of the yeast Rvs161 and Rvs167 protein domains. FEBS Lett 417: 21–27. 9395067 10.1016/s0014-5793(97)01248-9

[37] Colwill K, Field D, Moore L, Friesen J, Andrews BJ (1999) In vivo analysis of the domains of yeast Rvs167p suggests Rvs167p function is mediated through multiple protein interactions. Genetics 152: 881–893. 10388809 10.1093/genetics/152.3.881 PMC1460664

[38] Boone CJ, Bussey H, Andrews BJ (2007) Exploring genetic interactions and networks with yeast. Nat Rev Genet 8: 437–449. 17510664 10.1038/nrg2085

[39] Vallen EA, Caviston J, Bi E (2000) Roles of Hof1p, Bni1p, Bnr1p, and Myo1p in cytokinesis in Saccharomyces cerevisiae. Mol Biol Cell 11: 593–611. 10679017 10.1091/mbc.11.2.593 PMC14796

[40] Bi E (2001) Cytokinesis in budding yeast: the relationship between actomyosin ring function and septum formation. Cell Struct Funct 26: 529–537. 11942606 10.1247/csf.26.529

[41] Norden C, Liakoppoulos D, Barral Y (2004) Dissection of septin actin interactions using actin overexpression in saccharomyces cerevisiae. Mol Microbiol 53: 469–483. 15228528 10.1111/j.1365-2958.2004.04148.x

[42] Coll PM, Rincon SA, Izquierdo RA, Perez P (2007) Hob3p, the fission yeast ortholog of human BIN3, localizes Cdc42p to the division site and regulates cytokinesis. EMBO J 26: 1865–1877. 17363901 10.1038/sj.emboj.7601641 PMC1847667

[43] Kerppola TK (2008) Bimolecular fluorescence complementation (BiFC) analysis as a probe of protein interactions in living cells. Ann Rev Biophys 37: 465–487. 18573091 10.1146/annurev.biophys.37.032807.125842 PMC2829326

[44] Devrekanli A, Foltman F, Roncero C, Sanchez-Diaz A, Labib K (2012) Inn1 and Cyk3 regulate chitin synthase during cytokinesis in budding yeasts. J Cell Sci 125: 5453–5466. 22956544 10.1242/jcs.109157

[45] Ren G, Vajjhala P, Lee JS, Winsor B, Munn AL (2006) The BAR domain proteins: molding membranes in fission, fusion, and phagy. Microbiol and Mol Biol Rev 70: 37–120. 16524918 10.1128/MMBR.70.1.37-120.2006 PMC1393252

[46] Reyes A, Sanz M, Duran A, Roncero C (2007) Chitin synthase III requires Chs4p-dependent translocation of Chs3p into the plasma membrane. J Cell Sci 120: 1998–2009. 17519287 10.1242/jcs.005124

[47] Friesen H, Humphries C, Ho Y, Schub O, Colwill K, et al (2006) Characterization of the yeast amphiphysins Rvs161p and Rvs167p reveals roles for the Rvs heterodimer in vivo. Mol Biol Cell 17: 1306–1321. 16394103 10.1091/mbc.E05-06-0476 PMC1382319

[48] Brizzio V, Gammie AE, Rose MD (1998) Rvs161p interacts with Fus2p to promote cell fusion in *Saccharomyces cerevisiae* . J Cell Biol 141: 567–584. 9566960 10.1083/jcb.141.3.567 PMC2132759

[49] Xiong Y (2007) Study on cytokinesis and vesicle trafficking in budding yeast, *Saccharomyces cerevisiae*. Ph. D. thesis, Lancaster University.

[50] Nkosi PJ, Targosz B-S, Labib K, Sanchez-Diaz A (2013) Hof1 and Rvs167 Have Redundant Roles in Actomyosin Ring Function during Cytokinesis in Budding Yeast. PLoS One 8: e57846. 23469085 10.1371/journal.pone.0057846 PMC3585203

[51] Navarro P, Durrens P, Aigle M (1997) Protein-protein interaction between the RVS161 and RVS167 gene products of *Saccharomyces cerevisiae* . Biochim Biophys Acta 1343: 187–192. 9434108 10.1016/s0167-4838(97)00108-8

[52] Friesen H, Colwill K, Robertson K, Schub O, Andrews BJ (2005) Interaction of the Saccharomyces cerevisiae cortical actin patch protein Rvs167p with proteins involved in ER to Golgi vesicle trafficking. Genetics 170: 555–568. 15802519 10.1534/genetics.104.040063 PMC1450407

[53] Pinto IM, Rubinstein B, Kucharavy A, Unruh JR, Li R (2012) Actin depolymerization drives actomyosin ring contraction during budding yeast cytokinesis. Dev Cell 22: 1247–1260. 22698284 10.1016/j.devcel.2012.04.015 PMC3376349

[54] Wloka C, Vallen EA, Thé L, Fang X, Oh Y, et al (2013) Immobile myosin-II plays a scaffolding role during cytokinesis in budding yeast. J Cell Biol 200: 271–286. 23358243 10.1083/jcb.201208030 PMC3563683

[55] Sherman F (1991) Getting started with yeast. Methods Enzymol 194: 3–21. 2005794 10.1016/0076-6879(91)94004-v

[56] Gietz RD, Woods RA (2002) Transformation of yeast by the LiAc/SS carrier DNA/PEG method. Methods Enzymol 350: 87–96. 12073338 10.1016/s0076-6879(02)50957-5

[57] Longtine MS, McKenzie A, Demarini DJ, Shah NG, Wach A, et al (1998) Additional modules for versatile and economical PCR-based gene deletion and modification in *Saccharomyces cerevisiae* . Yeast 14: 953–61. 9717241 10.1002/(SICI)1097-0061(199807)14:10<953::AID-YEA293>3.0.CO;2-U

[58] Sheff MA, Thorn KS (2004) Optimised cassettes for fluorescent protein tagging in *Saccharomyces cerevisiae* . Yeast 21: 661–670. 15197731 10.1002/yea.1130

[59] Hentges P, Van Driessche B, Tafforeau L, Vandenhaute J, Carr AM (2005) Three novel antibiotic marker cassettes for gene disruption and marker switching in *Schizosaccharomyces pombe* . Yeast 22: 1013–1019. 16200533 10.1002/yea.1291

[60] Sung MK, Huh WK (2007) Bimolecular fluorescence complementation analysis system for in vivo detection of protein-protein interaction in *Saccharomyces cerevisiae* . Yeast 24: 767–775. 17534848 10.1002/yea.1504

[61] Phillipsen P, Stotz A, Scherf C (1991) DNA of *Saccharomyces cerevisiae* . Methods Enzymol 194: 169–181. 2005785 10.1016/0076-6879(91)94014-4

[62] Adams AEM, Pringle JR (1991) Staining of actin with fluorochrome conjugated phalloidin. Eds. Guthrie, C. and Fink, G.R. Methods Enzymol. 194: : 565–601. 10.1016/0076-6879(91)94054-g 2005819

[63] Krause SA, Cundell MJ, Poon PP, McGhie J, Johnston GC, et al (2012) Functional specialisation of yeast Rho1 GTP exchange factors. J Cell Sci 125: 2721–2731. 22344253 10.1242/jcs.100685

[64] Zhao H, Michelot A, Koskela EV, Tkach V, Stamou D, et al (2013) Membrane-Sculpting BAR Domains Generate Stable Lipid Microdomains. Cell Reports 4: 1213–1223. 24055060 10.1016/j.celrep.2013.08.024 PMC4105227

