# Supplementary figures and images for: The Budding Yeast Amphiphysin Complex Is Required for Contractile Actin Ring (CAR) Assembly and Post-Contraction GEF-Independent Accumulation of Rho1-GTP
Source: PLoS One. 2014 May 29;9(5):e97663. doi: 10.1371/journal.pone.0097663 (PMC4038553; doi:10.1371/journal.pone.0097663)

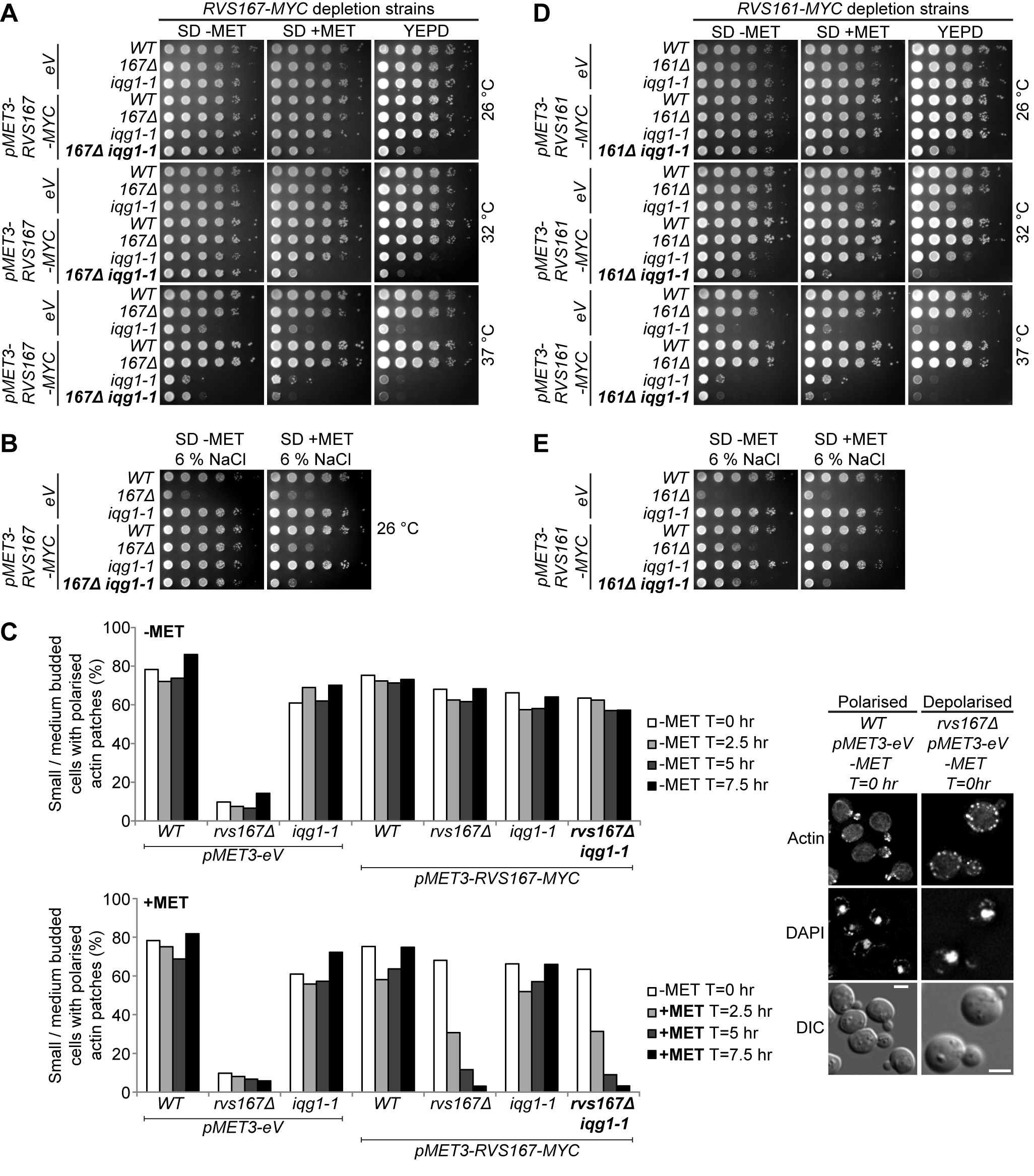

Supplement: Figure S1 — Rvs167-13Myc is functional. Indicated strains were grown to mid-log phasein media lacking methionine before plating onto indicated media and grown at temperature shown before imaging. A. Ectopic Rvs167-Myc expression rescues rvs167Δ iqg1-1 synthetic lethality (48 h growth). B. Ectopic Rvs167-Myc expression rescues salt sensitivity associated with rvs167Δ (128 h growth). C. Rvs167-Myc expression rescues actin polarity defects associated with rvs167Δ. Cells with ≥80% of actin patches localised in the bud or immediately adjacent at the bud neck were scored as polarised (between 94–145 cells scored at each time point). Shown are representative images from −MET (T = 0) of WT cells, with polarised actin patches, and rvs167 Δ cells with polarised (−MET) and depolarised (+MET) patches respectively. eV = ‘empty’ control vector. Scale bars = 2.5 um. D. Ectopic Rvs161-Myc expression rescues rvs161 Δ iqg1-1 synthetic lethality. E. Ectopic Rvs161-Myc expression only partially restores rvs161Δ salt sensitivity after 128 h growth. (TIF) [file pone.0097663.s001.tif]

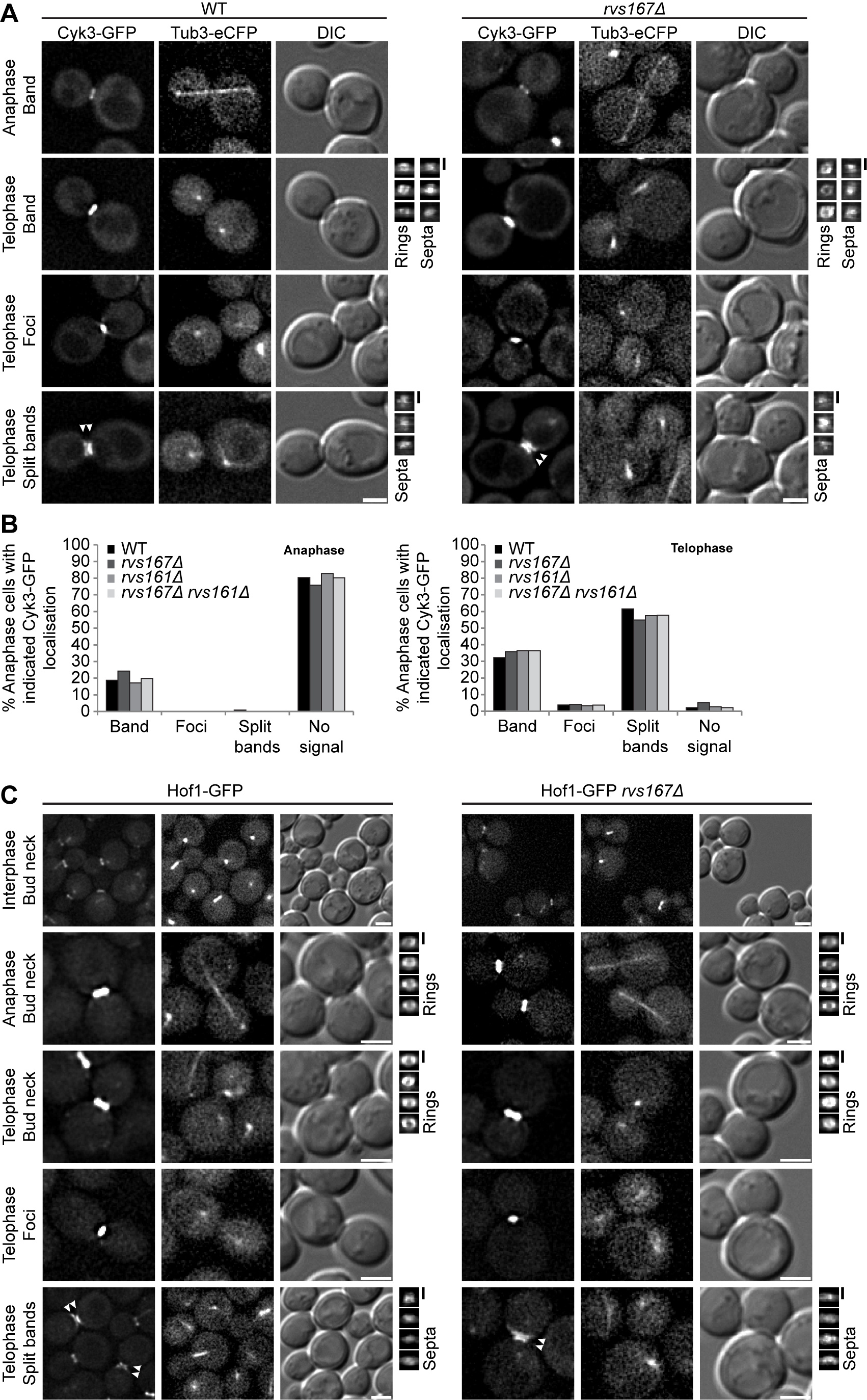

Supplement: Figure S2 — Cyk3 and Hof1 behaviour is unaltered in the absence of amphiphysin function. (A) CYK3-GFP localisation is unaltered in rvs167Δ cells as shown by representative average intensity fluorescent images (scale bars = 2 µm). Maximum projected reconstructions of pixel data at the bud neck demonstrate Cyk3-GFP localises at ring and septal structures (scale bars for reconstructions = 1 µm). (B) Quantification of Cyk3-GFP localization in large budded anaphase WT (n = 111), rvs167 Δ (n = 66), rvs161 Δ (n = 99), rvs167 Δ rvs161 Δ (n = 106) cells (left graph) and telophase WT (n = 130), rvs167 Δ (n = 173), rvs161 Δ (n = 148), rvs167 Δ rvs161 Δ (n = 135) cells (right graph). (C) Hof1-GFP localisation is unaltered in rvs167Δ cells. Fluorescent panels represent average intensity projections (scale bars = 2.5 µm), except bud neck reconstructions (maximum projections, scale bars 1 µm) that show Hof1-GFP localisation to rings in anaphase and telophase and to septa in late telophase. Cyk3-GFP (A) and Hof1-GFP (C) both form split bands either side of the division site in late telophase (arrowheads). (TIF) [file pone.0097663.s002.tif]

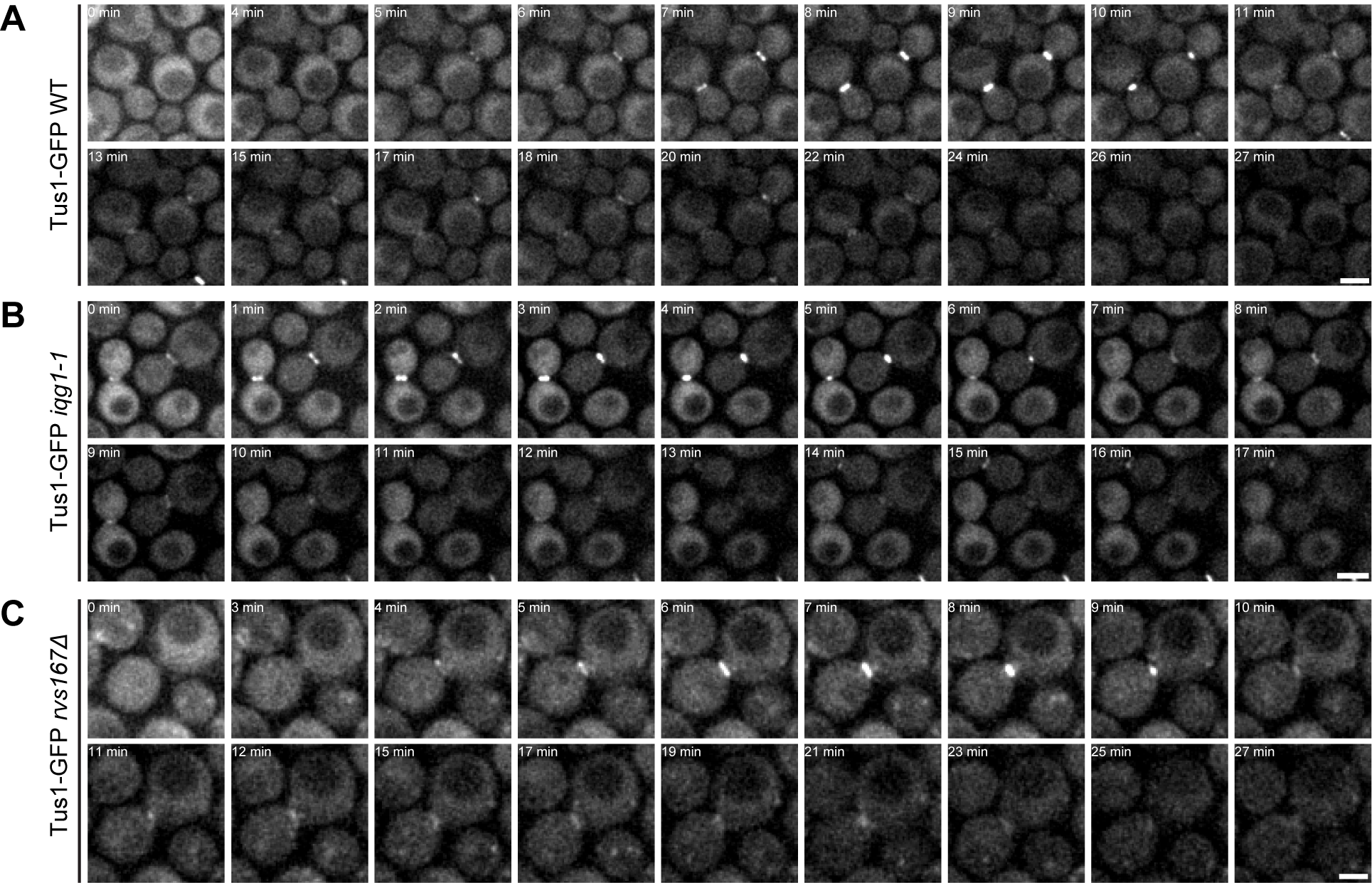

Supplement: Figure S3 — Tus1-GFP dynamics are normal in iqg1-1 and rvs167Δ mutants. WT (A), iqg1-1 (B) and rvs167 Δ (C) cells expressing Tus1-GFP were imaged at 1 minute intervals (3 second exposures, 18 z-sections, 0.2 µm z-spacing) for 35 minutes. Maximum intensity projections of deconvolved z-stacks are shown for indicated time points. (TIF) [file pone.0097663.s003.tif]
